# Supplementary material for: Topology optimization on metamaterial cells for replacement possibility in non-pneumatic tire and the capability of 3D-printing
Source: PLoS One. 2023 Oct 13;18(10):e0290345. doi: 10.1371/journal.pone.0290345 (PMC10575546; doi:10.1371/journal.pone.0290345)
Supplement: S1 Graphical abstract — (DOCX) [file pone.0290345.s001.docx]

**Graphical Abstract**

| \| Selected geometries for optimization \| Selected cells for comparison \| \| --- \| --- \| \| 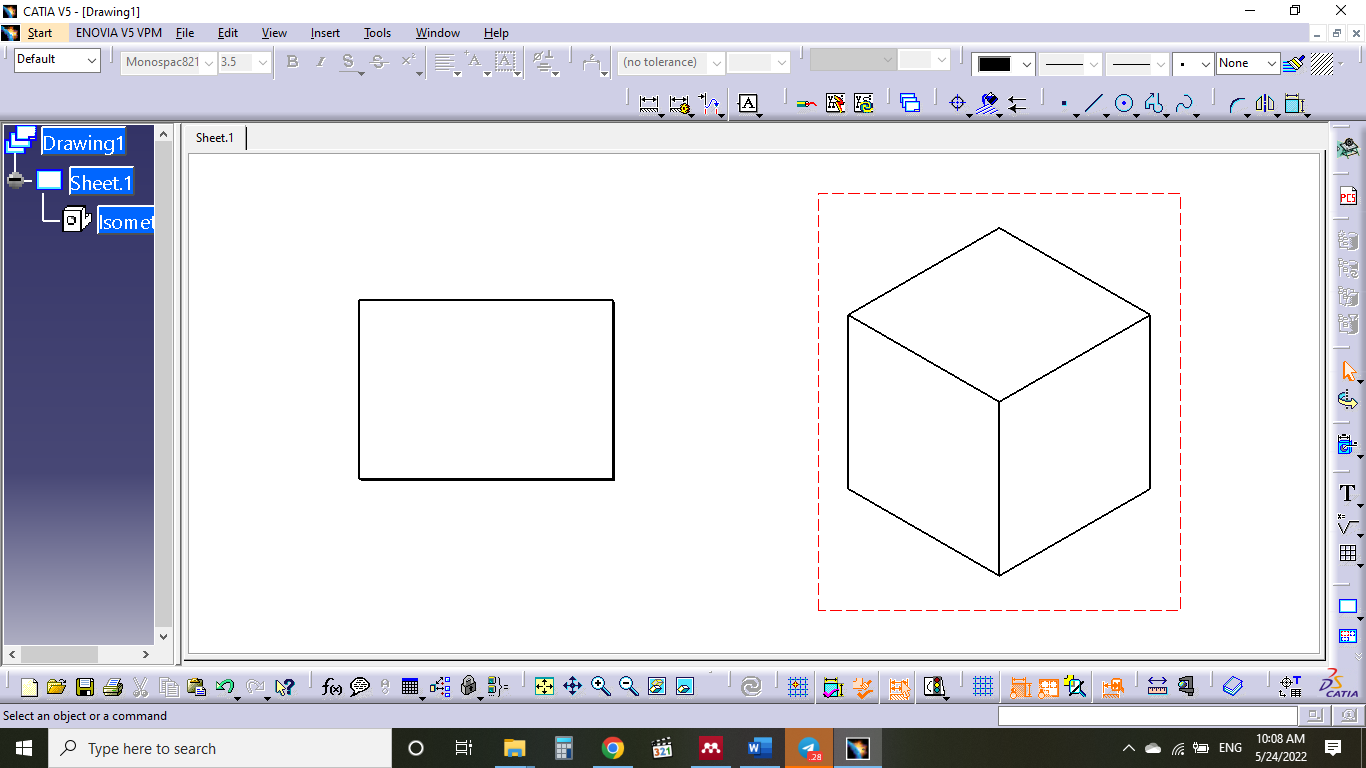 \| 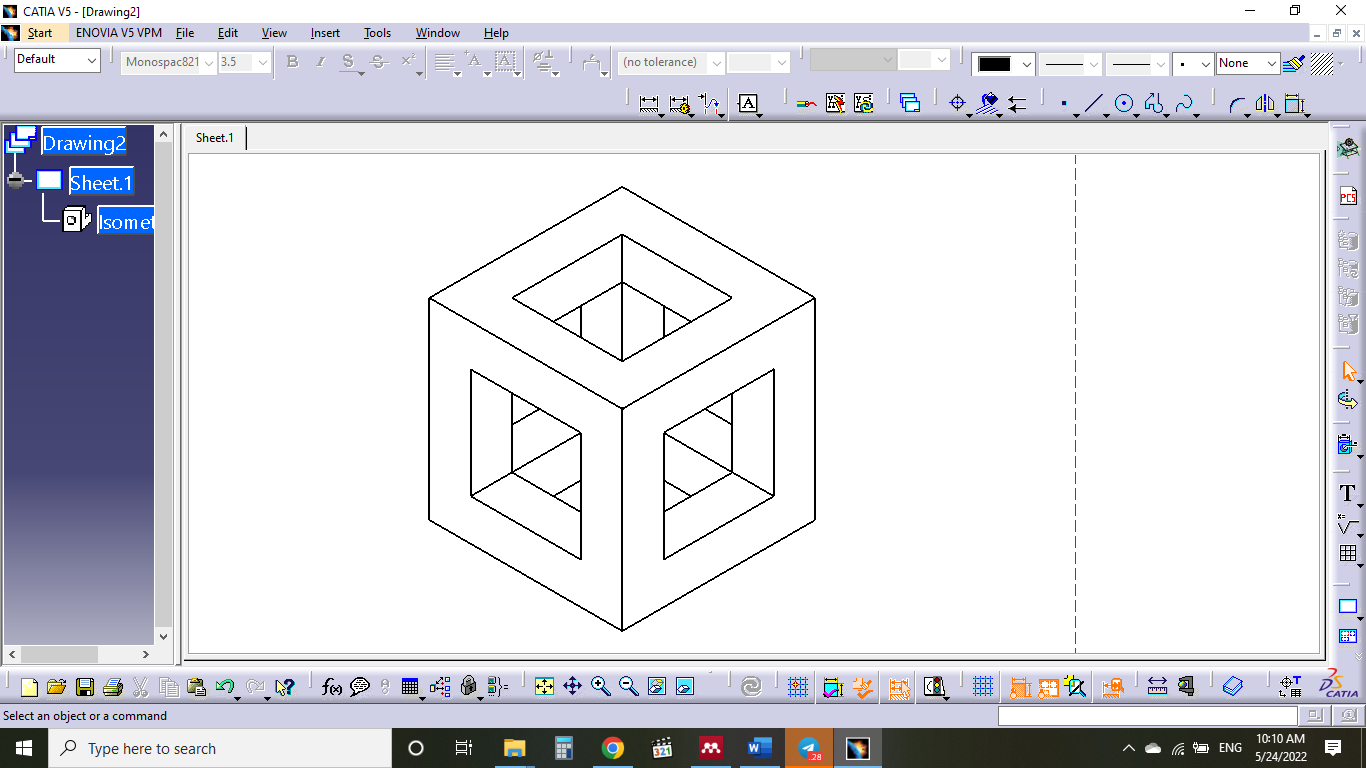 \| \| Cubic unit cell \| HC unit cell \| \| 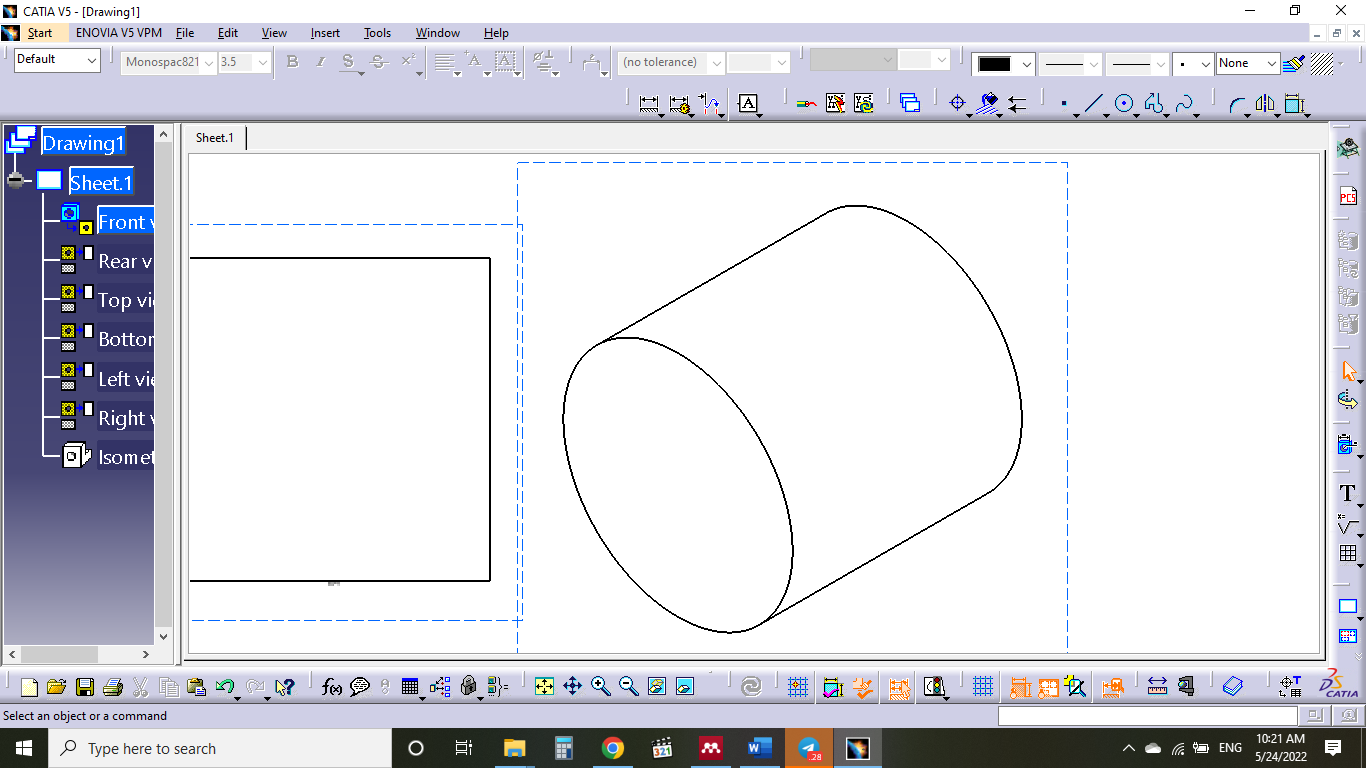 \| 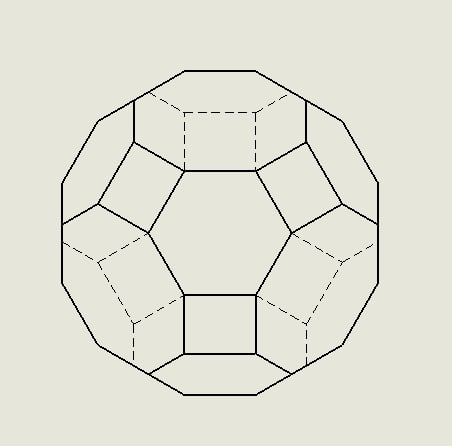 \| \| Cylindrical unit cell \| TC cell \| \| 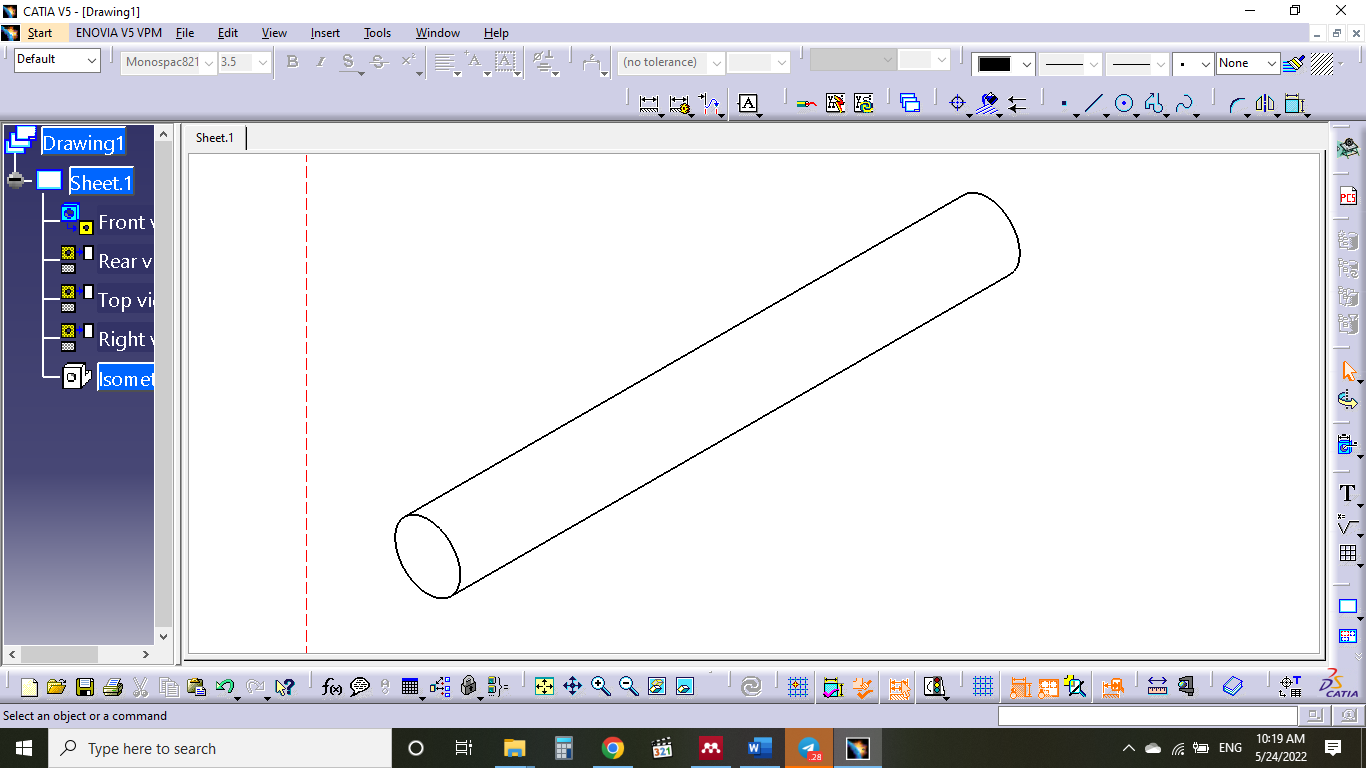 \| 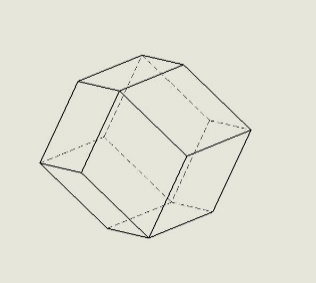 \| \| FTCS \| RD cell \| | **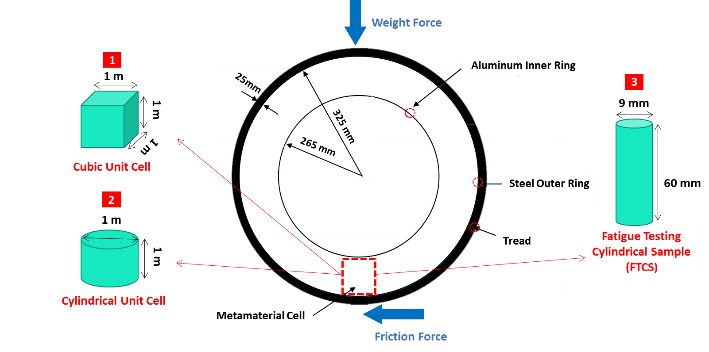**  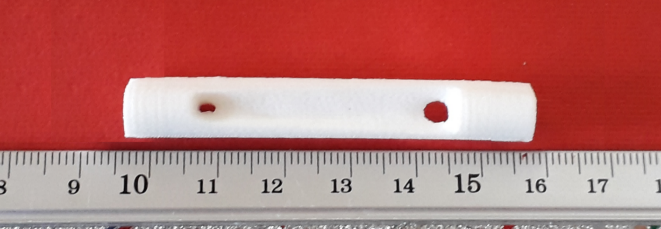  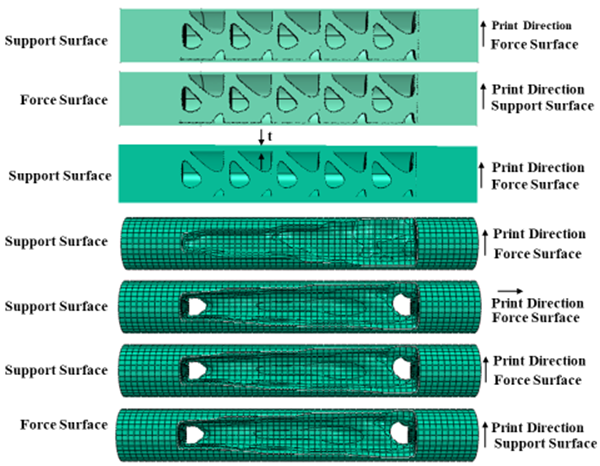  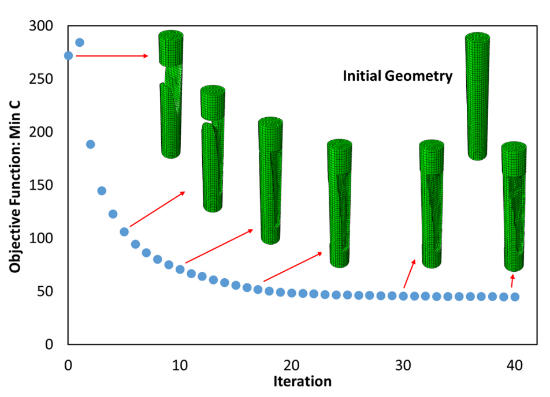 |
| --- | --- | --- | --- | --- | --- | --- | --- | --- | --- | --- | --- | --- | --- | --- | --- |
| 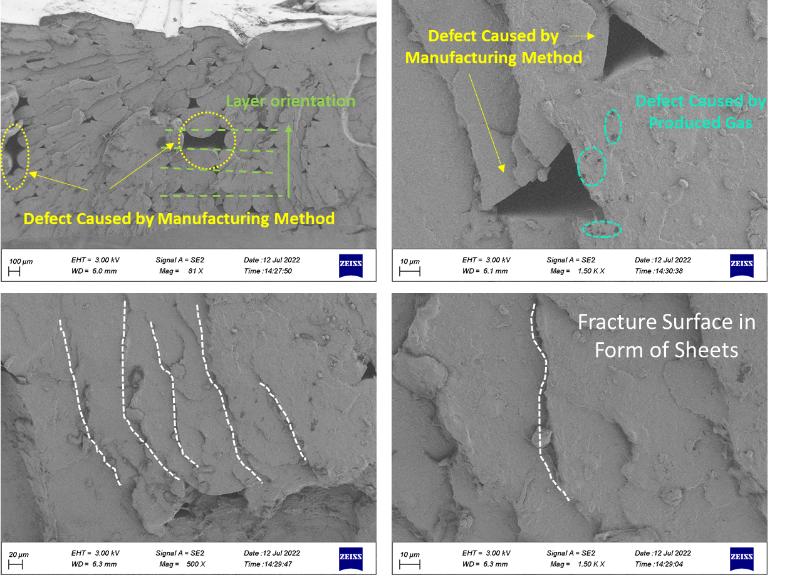 | 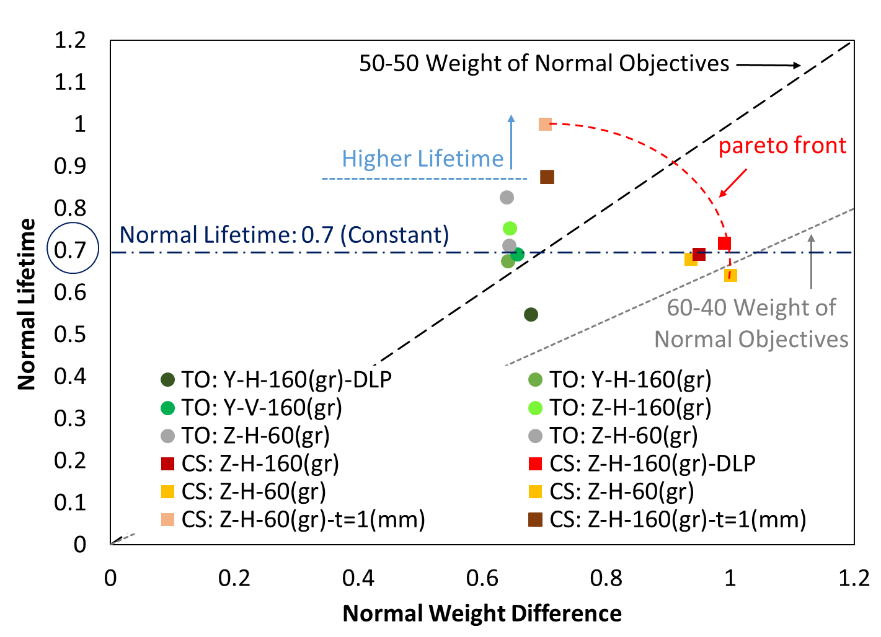 |
