# Supplementary material for: Topology optimization on metamaterial cells for replacement possibility in non-pneumatic tire and the capability of 3D-printing
Source: PLoS One. 2023 Oct 13;18(10):e0290345. doi: 10.1371/journal.pone.0290345 (PMC10575546; doi:10.1371/journal.pone.0290345)
Supplement: S1 File — (DOCX) [file pone.0290345.s002.docx]

**S1 File: A literature review on NPTs**

The companies that are active in the manufacture of NPTs are: Michelin, Goodyear, Uniroyal, Yokohama, Bridgestone, Britek, Toyo, Hancock, Polaris, Amerityre, Bigrep, Boeing, Kumho Tires, and Resilient Technologies that The introduction of manufactured tires and the activities carried out will be discussed [9].

Michelin is a tire manufacturer in France. This company has become unique by using Uptis technology which means anti-puncture for NPTs. These tires are safer since they do not break down and there is no need to change the tire. They also help the environment by reducing the raw materials needed [8].

In addition, Michelin has released another tire named Tweel. One of the prominent features of this tire is its lightness. These tires are suitable for all types of roads and their unique design causes better stability and reduces driver fatigue due to rebound effects. Moreover, they last 2 to 3 times longer than normal pneumatic tires. Tweel tires have been studied from various aspects to have the necessary and sufficient performance. Among the activities carried out regarding this tire is the ability to make them with 3D printers. This research was done by Suvanjumrat and Rugsaj [12]. In this research, the standard tensile test samples were prepared once with the help of a 3D printer and again with the help of a water jet from Tweel tire cells and compared with each other. With the obtained results, a simulation has been made and its results have been compared with the tire produced by the 3D method. The results, which include the maximum tension and vertical stiffness, show that the tire produced by the 3D printer method is not different from the original tire. But considering the ability of 3D printers to produce complex shapes, this method can be more useful. In another study [13] the aforementioned researchers studied the mechanical behaviors of Tweel tires to develop a dynamic finite element model. The material properties in this model are compared with the tensile and compressive test results on the real NPT sample. The results have shown a good agreement between the finite element model and the experimental results. Moreover, the performance of NPT on flat roads was analyzed.

In addition, the three-point bending test and the simulation of this test with the help of the finite element method have also been performed by these researchers [16]. In another study, these researchers studied the effects of cell thickness on vertical stiffness, maximum local stress in cells, and their weight with finite element modeling and tests on a real tire. Different loads were applied to the tire and tensile states were observed in the upper part of the tire and bending states in the lower part of the cells. The results showed that the thickness of 5 mm is a suitable thickness for the purposes considered [17]. In addition to this, another research has been conducted on the noise produced by Tweel tires. This tire produces an acoustic sound during rotation at high speed, which is assumed to be due to the resonant vibration of the tire cells when they enter the contact area and the cells buckle and then return to the tension state. To identify and understand the causes of sound noise for this tire, a nonlinear finite element model has been created using Abaqus. This model has studied the changes in cell thickness and tire rotation speed on the vibration amplitude and frequency of the cells [18]. Manibaalan et al. [19] have studied different parameters affecting rolling resistance and also different cross-sections of Tweel tires. It has been concluded that since the Tweel tire is made of polyurethane and polyurethane has both stiffness and flexibility, this tire can perform better than pneumatic tires. In addition, by changing the geometry of the structure or changing the properties of the polyurethane-based composite, different loads and different applications can be expected from this type of tire. Moreover, the rolling resistance of this tire is lower than pneumatic tires, and as a result, fuel consumption should be reduced. Zmuda et al. [20] also presented a numerical model to predict the pressure distribution in the cells of this tire.

One of the goals of Michelin company is to use Tweel tire on the moon. Therefore, a research has been done in this regard and the performance of the tire in the face of sand has been investigated from different aspects, which led to a new structure of Tweel tire called Tweel^TM^. has been Ma et al. [21] have used the finite element method to model and simulate the dynamic interaction between tire and sand. Numerical results show that the deformation of the tire at a lower speed is much higher than at a higher speed. However, at higher speeds, the higher contact pressure is created and the resulting contact pressure is not uniform. Moreover, higher movement speed in sand produces higher values ​​of the von-Mises stress and residual plastic strains than lower movement speed.

In addition to the tire itself, the interaction of the tire shear layer with sand has also been studied by these researchers. In this research, the shear layer is considered a cellular and metamaterial structure, and the geometric optimization of this cell has been discussed. The new design can withstand cyclic loads and extreme temperatures from 40 to 400 K. For this reason, linear elastic metals have been used instead of polyurethane in the shear layer. Another goal of this project is to reduce tire rolling resistance by 50% while maintaining structural compliance in the elastic range under pressure in the contact area between the tire and the road [22]. Some activities have been carried out regarding finding a suitable pattern for the cutting layer. Among others, we can mention the research of Shankar et al. [23]. They studied three different types of patterns to find a cell with a high shear bending force bearing in mind the strain energy distribution. In addition, Ma et al [14] investigated several ribbed metamaterial shear layers and determined the shear layer with a specific cellular geometry with the best pressure distribution in the contact area. Then these researchers optimized the size of this cell and found the optimal thickness of the supporting layers of these cells. In another research, to better understand the performance of Tweel^TM^ with sand and provide information for further optimization of this tire, the interaction between tire and sand has been done using the finite element method of modeling and simulation [15].

Despite all the research done, Michelin is working on another NPT that is fully compatible with the environment and has sensors to collect real information from the tire. The main idea of these tires is the ability to 3D print different patterns on the tire tread depending on the working conditions of the tire [8].

Goodyear company has introduced tires that are produced from recycled powder from old tires and are completely compatible with the environment. These powders are used as a raw material in tire production through a 3D printer with selective laser sintering (SLS) process. Another feature of these tires is the absorption of carbon dioxide and the exhalation of oxygen [8].

Taking inspiration from nature, Kumho Tires has introduced a type of tire called BON, which means born in nature.

Bigrep is a company that produces 3D printers. However, he produced a bicycle completely with a 3D printer method. In this bicycle, the tires are produced with filament made of TPU, which is durable and highly resistant to heat. In this bicycle, a honeycomb pattern is used to produce tires.

Nexen Tire Company has introduced a flower-like NPT called Dandelion. This tire has 72 pins that independently move up and down vertically based on the road condition, this arrangement allows the tire to be used on any uneven ground.

Bretek Company has also introduced another type of tire that converts the elastic potential energy stored in the wheel into useful forward movement.

Another type of NPT is called ME-Wheel. This tire can work like a pneumatic tire. One of the features of this tire is that it does not puncture and absorbs shocks. The structure of the wheel includes a core, metamaterial cells in the form of hinges, elastic rings, return springs, tread, and pin. The function of the springs is to generate tensile force to restore the closed hinges to their original state. The hinges do not undergo bending deformation in the contact area. These hinges are uniformly distributed in the radial direction and connect the core and tread of the tire and also have the task of bearing the axial load applied in the center of the core. Hinges can be placed next to each other in different ways and create a wide spectrum of ride comfort and stability in the car. Another noteworthy point of this tire is its tread. The pattern on the tread is designed in a way that reduces wear as much as possible. Moreover, the tread of the tire can be replaced in case of wear [28,29].

Youqun et al. [25] have considered two different types of hinge structures to investigate the effect of hinge arrangement on static stiffness. One structure consists of two joints and another structure consists of three joints in one hinge. Based on this, 4 types of tires have been designed. Their difference is in the structure and number of hinges.

Wei et al. [24] have investigated the vibration performance of this tire in the face of impact. They have considered non-linear factors such as geometry, material, and contact to create the finite element model. Vehicle vibration performance with MEW tires and a type of radial tire have been studied. The results show that the car with MEW tire meets the requirements of vibration performance and order of vibration performance of a pneumatic tire.

Zhao et al. [26] studied the MEW tire dynamically. It has been concluded that the rolling speed can have a great effect on the dynamic characteristics of this tire, and a higher sliding speed leads to higher vibration. The results of the practical test have been in good agreement with the results of finite element simulation. Deng et al. [27] also used a 3D nonlinear finite element model to numerically investigate this tire in a steady state. The stress in the important components of the tire, such as the ring and hinges, are analyzed based on the simulation results. It has been concluded that the developed model can predict the dynamic behavior of tires in different conditions with high reliability. It has been shown that the normal pressure distribution in these tires is completely different from pneumatic tires and the maximum stress occurs in the central area of ​​the contact surface, which improves tire resistance.

Du et al. [28] have studied MEW tires with different camber angles experimentally and using finite element simulation. These researchers have investigated the effect of camber angle on vertical stiffness, tire deformation, and pressure distribution on the contact surface [29]. Xiao et al. [30] have studied the durability of this tire to improve its lifetime. Moreover, the durability of the tire has been taken into account using the finite element method based on the virtual static ground created by the simulation and the reliability factor to calculate the tire lifetime.

Machining and installation errors are unavoidable in mechanical structures. Zhao et al. [31] investigated the effect of these types of errors on the radial hardness of MEW tires. For this purpose, the mathematical model and the finite element model for the tire have been created and compared with the results of the experimental test.
