# Supplementary material for: Topology optimization on metamaterial cells for replacement possibility in non-pneumatic tire and the capability of 3D-printing
Source: PLoS One. 2023 Oct 13;18(10):e0290345. doi: 10.1371/journal.pone.0290345 (PMC10575546; doi:10.1371/journal.pone.0290345)
Supplement: S2 File — (DOCX) [file pone.0290345.s003.docx]

**S1 File: A benchmark on cells and their properties**

Reviewing articles to find the cells with the best mechanical properties was done. In Table B1 and Figure B1, these cells with their properties are shown. In addition, Figure B2 shows the application for these cells.

Table B1: Different types of cells and their properties

| Materials | Load types | Fabrication methods | Results | Ref. |
| --- | --- | --- | --- | --- |
| PLA | Tension- Compressive fatigue | FFF | Unit cell shape, direction, type, and amount of mechanical loading have significant effects on material properties. | [40] |
| TPU | Compressive Fatigue | SLS | The auxetic properties are preserved in a wide range of applied deformations. | [41] |
| PETG | Uniaxial tension | FFF | 3D printed metamaterial samples show much higher strains than standard 3D printed tensile samples. | [52] |
| Shape memory polymer (SMP) | thermal | 4D-FFF | Self-locking property has been observed in the sample. | [63] |
| PLA | Uniaxial compression | 4D-FFF | The metamaterial with auxetic unit cells has a better energy absorption capability compared to other structures studied in this article. | [68] |
| PLA | compressive and impact | FFF | The elastic modulus is increased.  In the impact test, it has been shown that the angle of the cell does not have much effect on the results. | [69] |
| Poly carbonate | Noise and vibrations | FFF | In addition to reducing the mass of the system, the designed metamaterial can be used to absorb low-frequency vibration and wave guidance. | [70] |
| Resin | Tension and compressive | Stereolithography (SLA) | - Stress concentration is reduced compared to other metamaterials.  - This cell has a negative Poisson ratio in a wide range. | [71] |
| Aluminum 6060 with heat treatment | Tensile fatigue | CNC | Fatigue lifetime in auxetic samples has increased by 20% compared to samples with a positive Poisson ratio. | [72] |
| SMP | Axial tension | 3D print | - This cell can reduce axial tension during expansion due to zero Poisson ratio.  - A stent with a smaller radius has higher critical stress and less displacement. | [73] |
| Polyamide 12 | Tensile and tensile fatigue | SLS | - CC cell has improved stiffness and fatigue lifetime and reduced stress concentration.  - CC cell has the lowest stress concentration coefficient.  - CC cell is more rigid than other cells. | [42] |
| Ti6Al4V | Compressive fatigue | Selective laser melting (SLM) | Increasing porosity has decreased fatigue resistance. | [43] |
| Ti6Al4V | Fully reversible bending fatigue  and tension-compression fatigue | SLM | Defects in the manufacturing process have reduced the fatigue lifetime, but do not affect elastic modulus. | [44] |
| AlSi7Mg | Compression and bending fatigue | SLM | - The total fatigue lifetime is divided into three stages: initiation of damage, and propagation on one or more bases leading to final failure.  - In fatigue loading, crack propagation has occurred in one or more foundations. | [45] |
| Ti-6Al-4V | Tension fatigue | EBM | - The shortened cube cell has endured the most compressive force.  - The yield stress in all cells is within the permissible range.  - The cubic cell has the highest mechanical properties. However, it also has the lowest Poisson ratio values.  - The diamond cell and RD cell have endured the least bending force. However, they have the highest Poisson ratio. | [46] |
| Chrome-cobalt | Fatigue | SLM | - The effect of the type of material on the fatigue properties is greater than the topological design.  - TC cell has the longest fatigue lifetime.  - The effect of manufacturing and topological design defects is different in high and low cycle fatigue areas. | [47] |
| Ti6Al4V ELI | Fatigue | SLM | - Static mechanical properties and fatigue properties of metamaterials depend on the type of unit cell and also on porosity.  - Higher porosity leads to shorter fatigue lifetime for the same level of applied stress.  - Cubic, TC, and diamond cells have the longest lifetime, respectively. | [48] |
| AlSi10Mg | Compressive | SLM | - Stress concentration around nodes is predicted using a model. | [49] |
| Ti6Al4V | - | SLM | - The effect of the production process and the direction of 3D printing on cell dimensions and the difference between the designed CAD file and the 3D printed part have been investigated.  - The radius of the fillet depends on the CAD file, the thickness of both the bases on which it is located, and the direction of the 3D print. | [50] |
| Ti6Al4V | Fully Reverse Fatigue | SLM | - Elastic modulus and fatigue resistance are related to the number and severity of defects.  - The material distribution does not only depend on the designed geometry but also on the 3D printing parameters.  - The bases with a low angle compared to the 3D printing plate have increased the thickness and reduced the stress concentration.  - The greater thickness of the bases has increased the elastic modulus, while the displacement of the connection center has decreased the elastic modulus.  - Fatigue resistance is more sensitive to surface irregularities such as sharp cuts in base connections than internal porosity. | [51] |
| Ti6Al4V ELI | Tensile fatigue | EBM | - Fatigue failure of cellular structures is gradual.  - Fatigue properties of cells depend on defects caused by incremental production. | [53] |
| 316 L stainless steel | Compressive | SLM | - The formation of plastic hinges in the bases near the nodal areas is shown.  - Hardness and yield strength can be improved by changing the geometry of the unit cell. | [54] |
| - | Thermal Fatigue | - | - auxetic structures have a longer lifetime than non- auxetic structures.  - Reducing the relative density of cells has increased the useful lifetime of structures. | [55] |
| Ti6Al4V | Compressive and torsional | EBM | - Cubic structures have more resistance and hardness than BCC and cross structures when the load is applied in a compressive manner.  - The mechanical properties for twisting in cross structures are better than in cubic structures. | [56] |
| Iron | corrosion fatigue | SLM | - Porous iron has shown high fatigue resistance.  - Fatigue strength is 70% and 65% of yield stress in the air and modified simulated body fluid (r-SBF), respectively. | [57] |
| IN718 | Tensile and tensile fatigue | SLM | - The tensile mechanical behavior of the cell structure is much lower than that of dog bone samples with the same parameters.  - The failure analysis showed that the cracks started at the intersection between the ligaments and nodes, which is the place of the highest stress concentration from the finite element analysis. | [58] |
| Ti6Al4V | Compressive fatigue | EBM | Elastic modulus and fatigue stress in the cubic cell are higher than in other cells. | [59] |
| Ti6Al4V | Compressive | SLM | - The SC cell has the highest elastic modulus and yield strength at any relative density, while the BCC cell is the lowest.  - The fatigue characteristic of cells depends on the relative density and topology of the single cell.  - Fatigue behavior of SC and SC-BCC cells is independent of relative density. | [60] |
| Ti6Al4V | Compressive Fatigue | SLM | - Cells have improved resistance to compressive fatigue due to the lack of stress concentration.  - Internal defects have been observed in the cell walls that are the starting point of cracks and fractures.  - Modifying the parameters of 3D printing has led to an increase in the lifetime of the sample. | [61] |
| Ti6Al4V | Fatigue | SLM | Removing the fillers significantly improves the S-N curves and the direction of the 3D printing affects both the fatigue strength and the fracture behavior. | [62] |
| Metal | compressive | SLS | Each cell exhibits a distinct failure mechanism that is controlled by topology and geometrical defects caused by additive manufacturing. | [64] |
| Ti2448 | Compressive and fatigue | EBM | - The elastic property is improved by increasing the porosity of the samples due to the increase in the tension/compression ratio. | [65] |
| Ti6Al4V | Compressive | SLM | - The elastic modulus and strength are correlated with the density and this performance is remarkably good despite the high roughness and irregularity of the foundations at this scale.  - Sudden surrender is shown visually. | [66] |
| Ti6Al4V | Tensile-tensile fatigue, compression-compression fatigue, and tension-compression fatigue | SLM | - Overall loading in tension-tension leads to a decrease in fatigue performance compared to overall loading in compression-compression.  - Compressive-compressive and tensile-tensile fatigue loadings lead to a shorter fatigue lifetime compared to completely reversible loading due to the presence of moderate local tensile stress. | [67] |

| 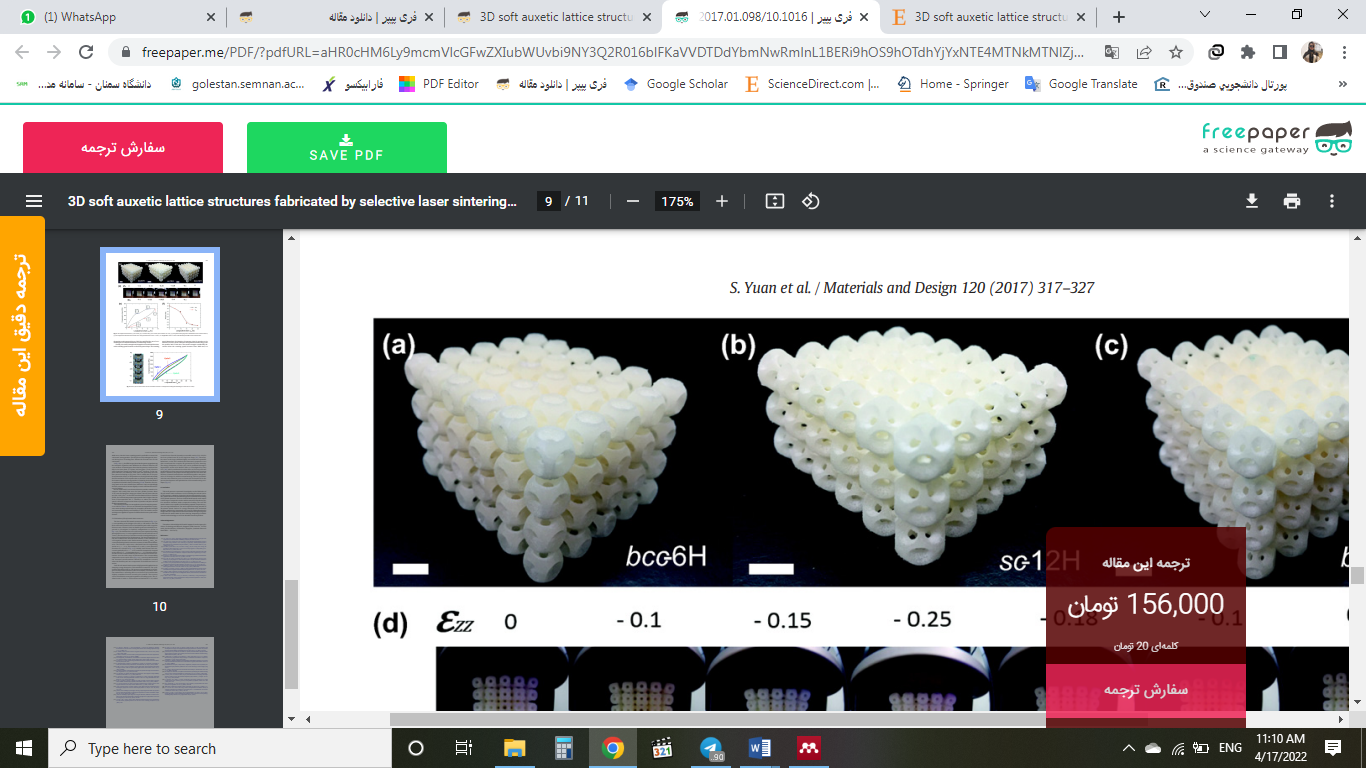 | Ref. [41] | 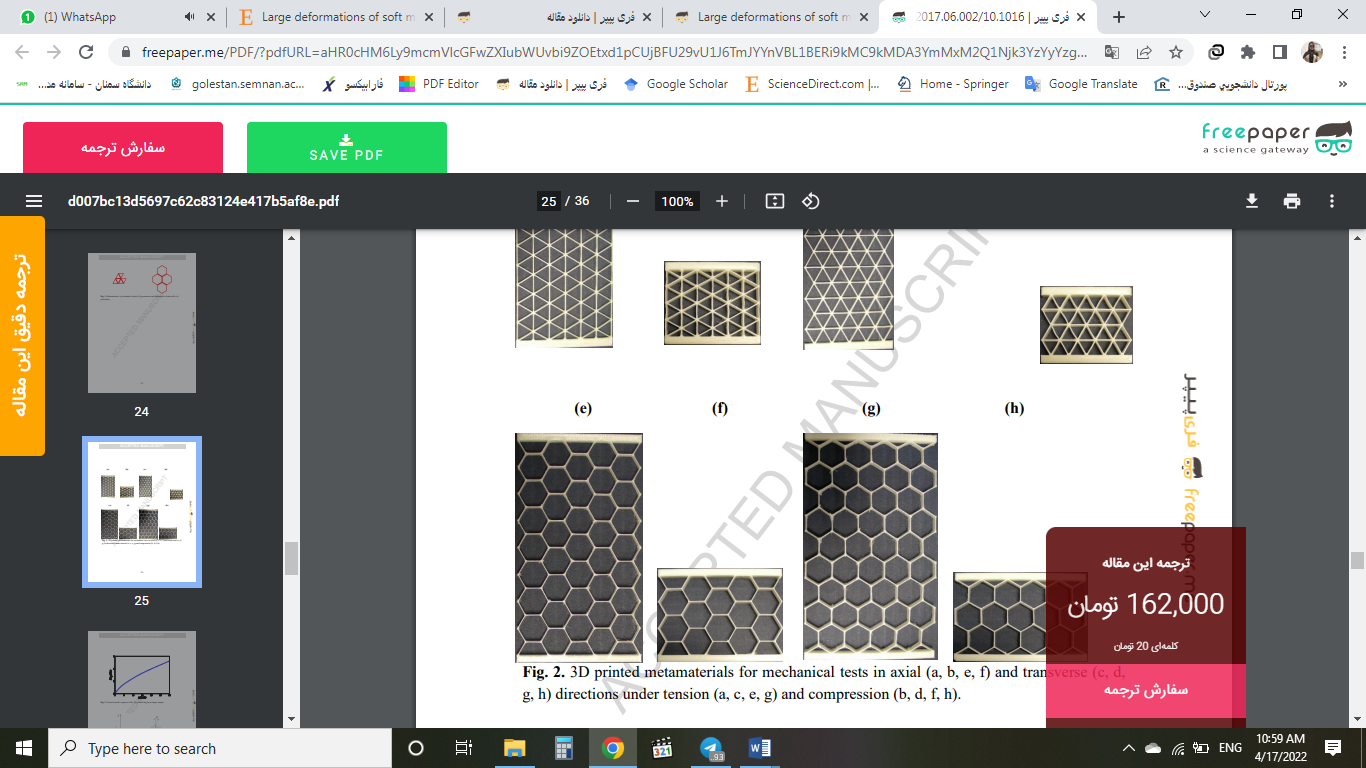 | Ref. [40] |
| --- | --- | --- | --- |
|  | Ref. [71] | 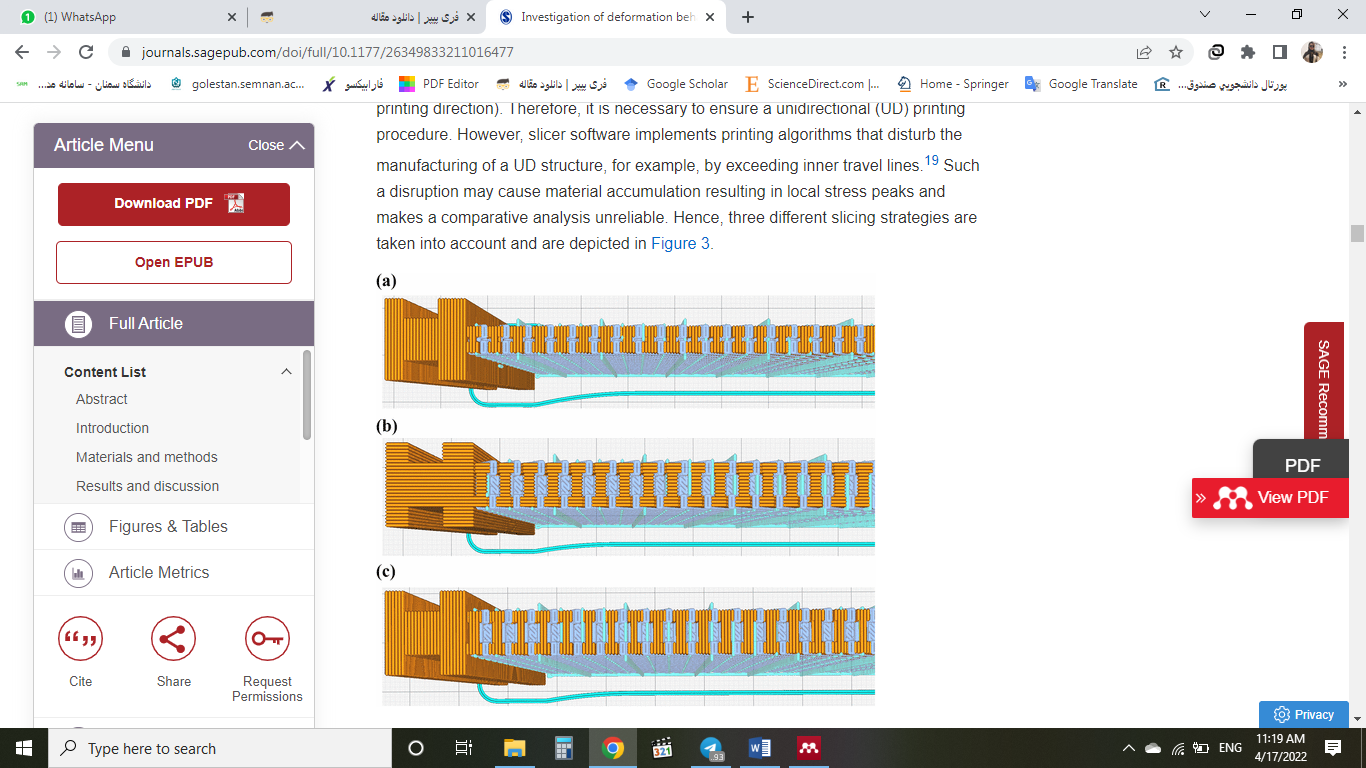 | Ref. [52] |
| **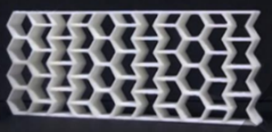**  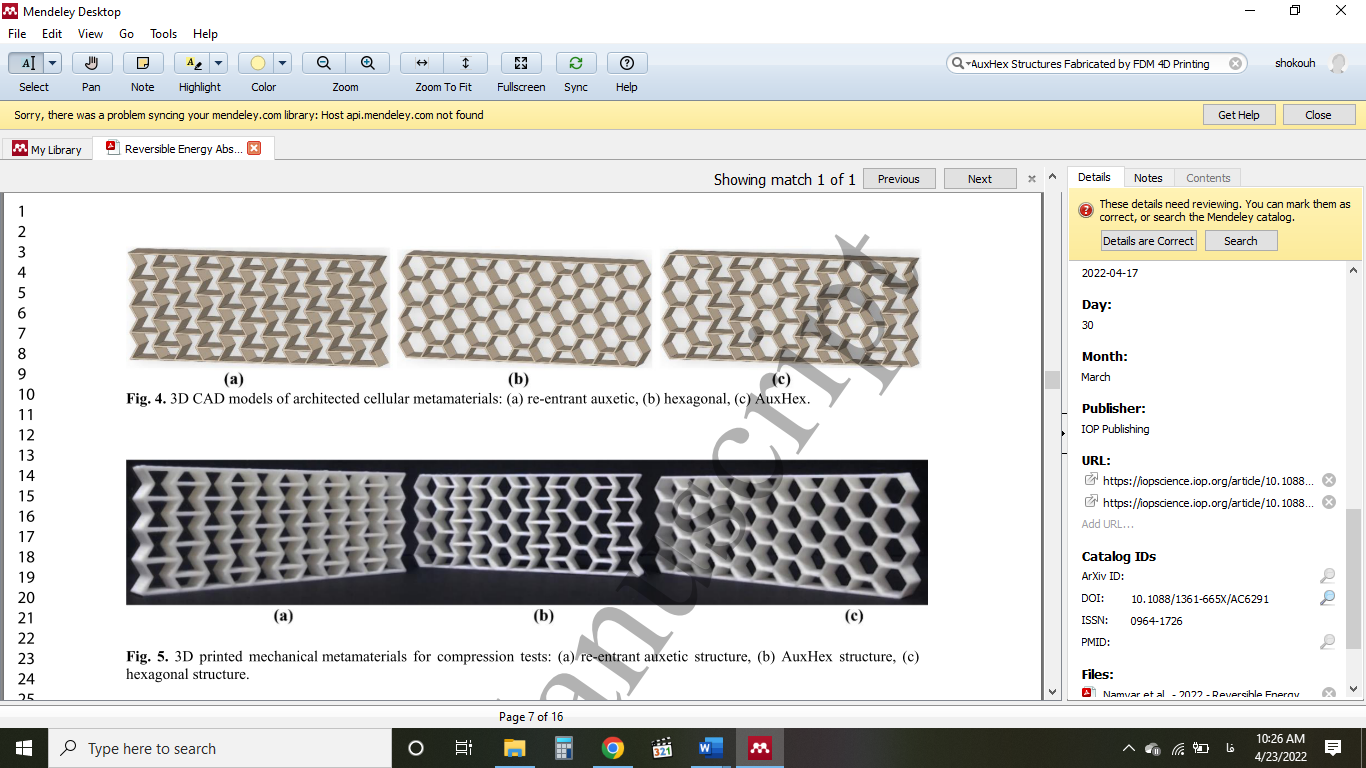 | Ref. [68] | **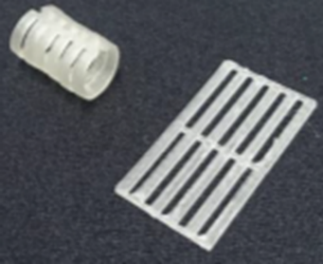** | Ref. [63] |
| **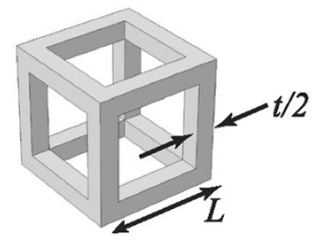** | Ref. [70] | **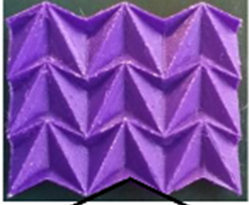** | Ref. [69] |
|  | Ref. [73] | 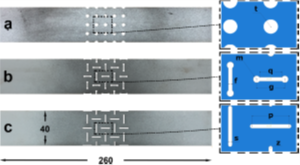 | Ref. [72] |
| 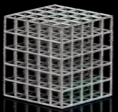 | Ref. [46] | 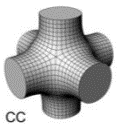 | Ref. [42] |
| 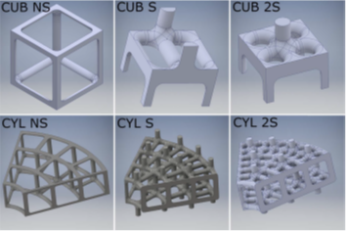 | Ref. [44] |  | Ref. [43] |
|  | Ref. [55] |  | Ref. [45] |
| 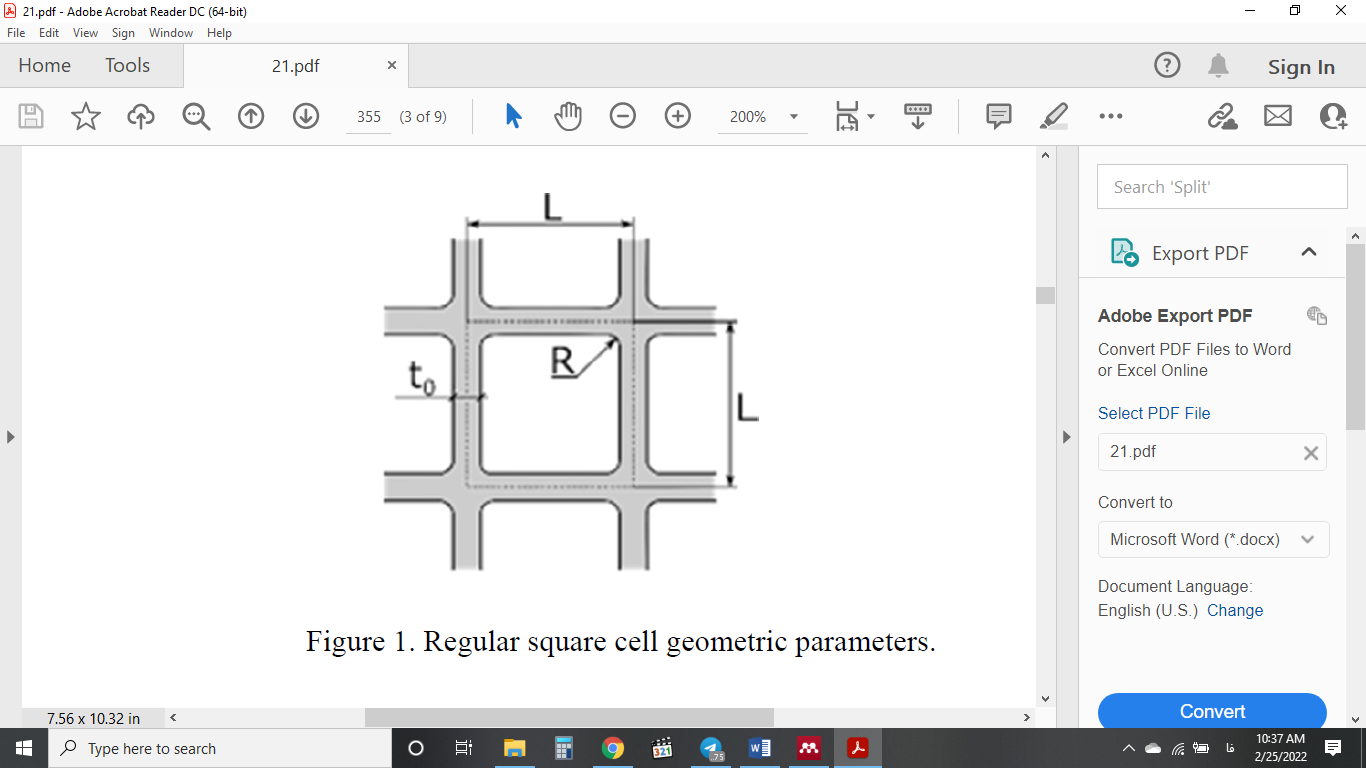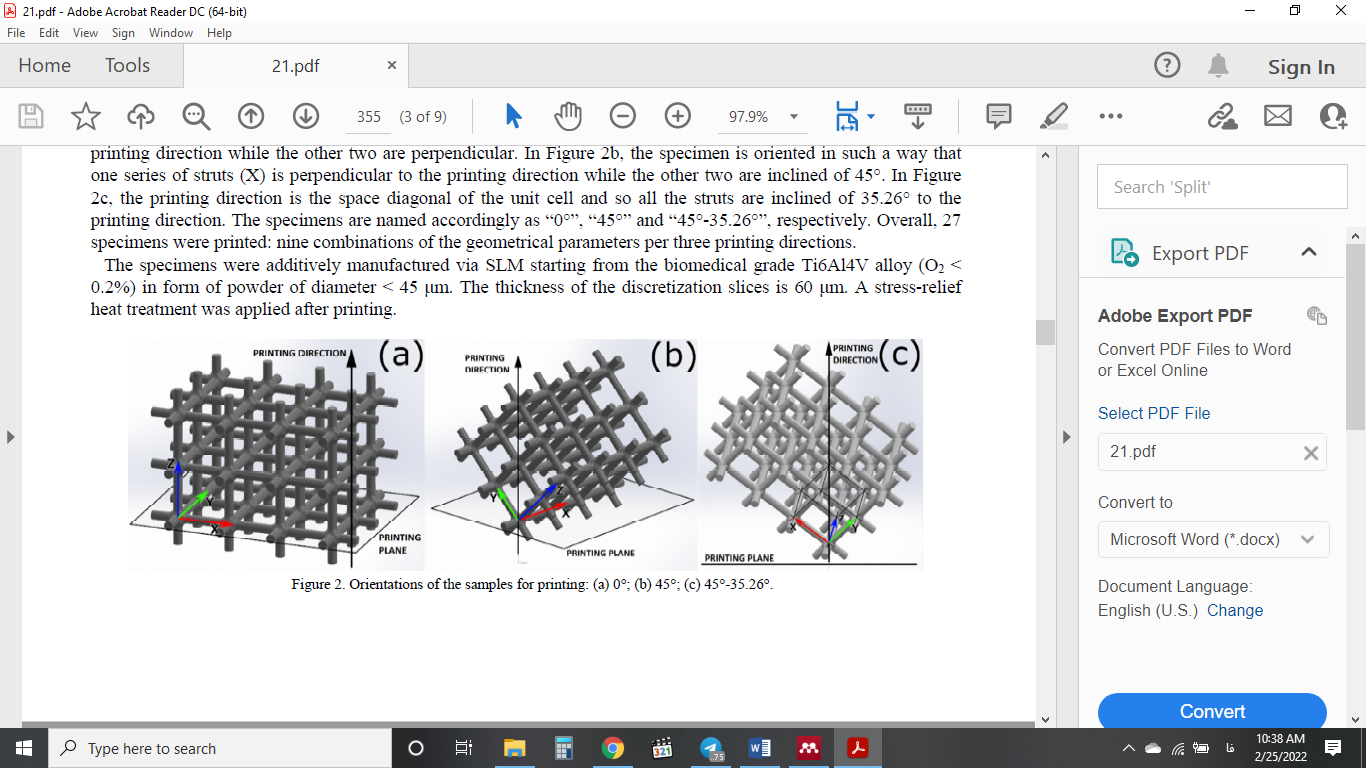 | Ref. [50] | 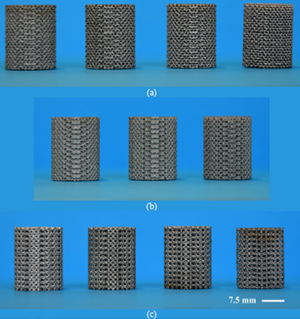 | Ref. [47] |
|    | Ref. [51] |  | Ref. [49] |
| 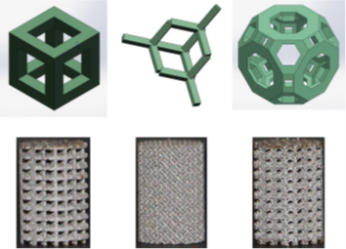 | Ref. [48] | 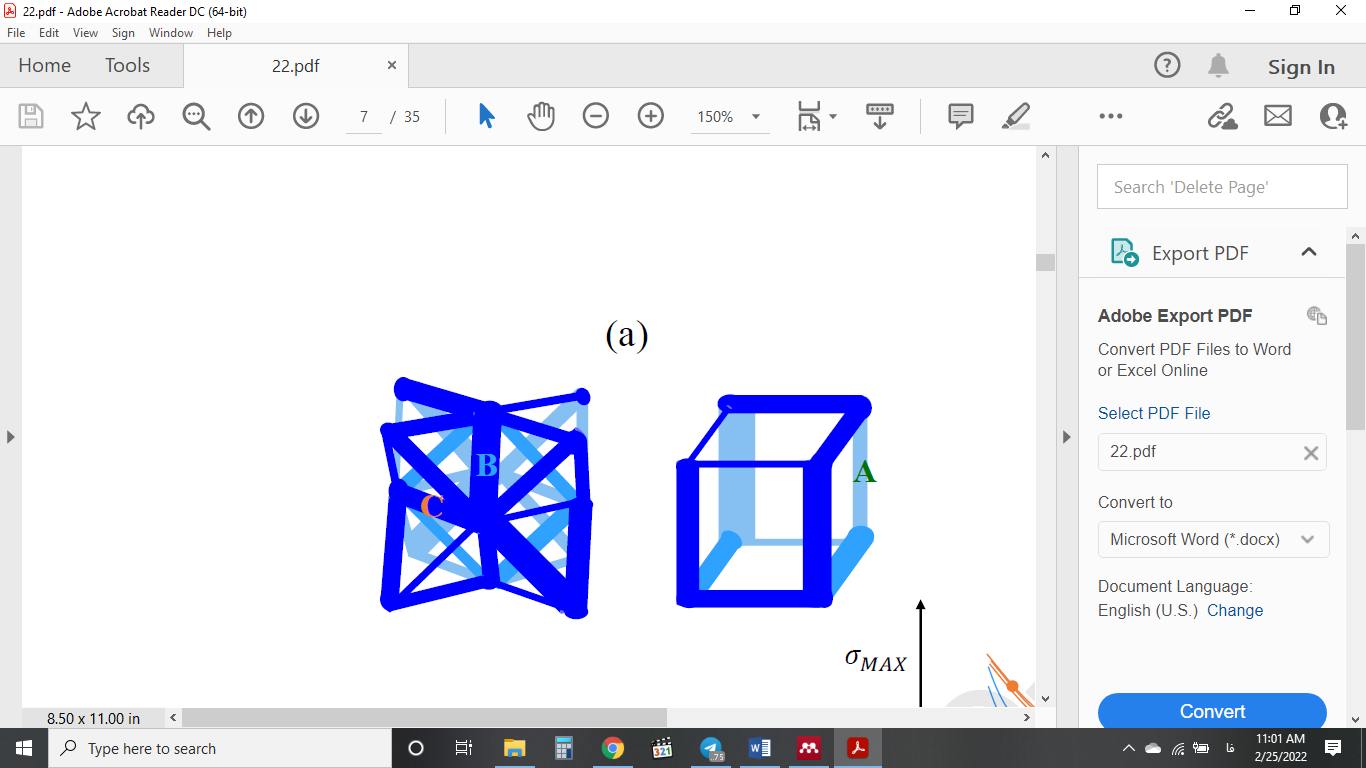 | Ref. [53] |
|  | Ref. [56] |  | Ref. [54] |
|  | Ref. [58] | 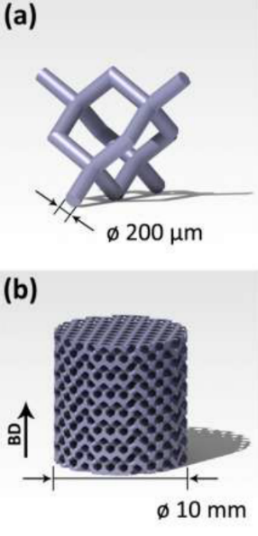 | Ref. [57] |
| 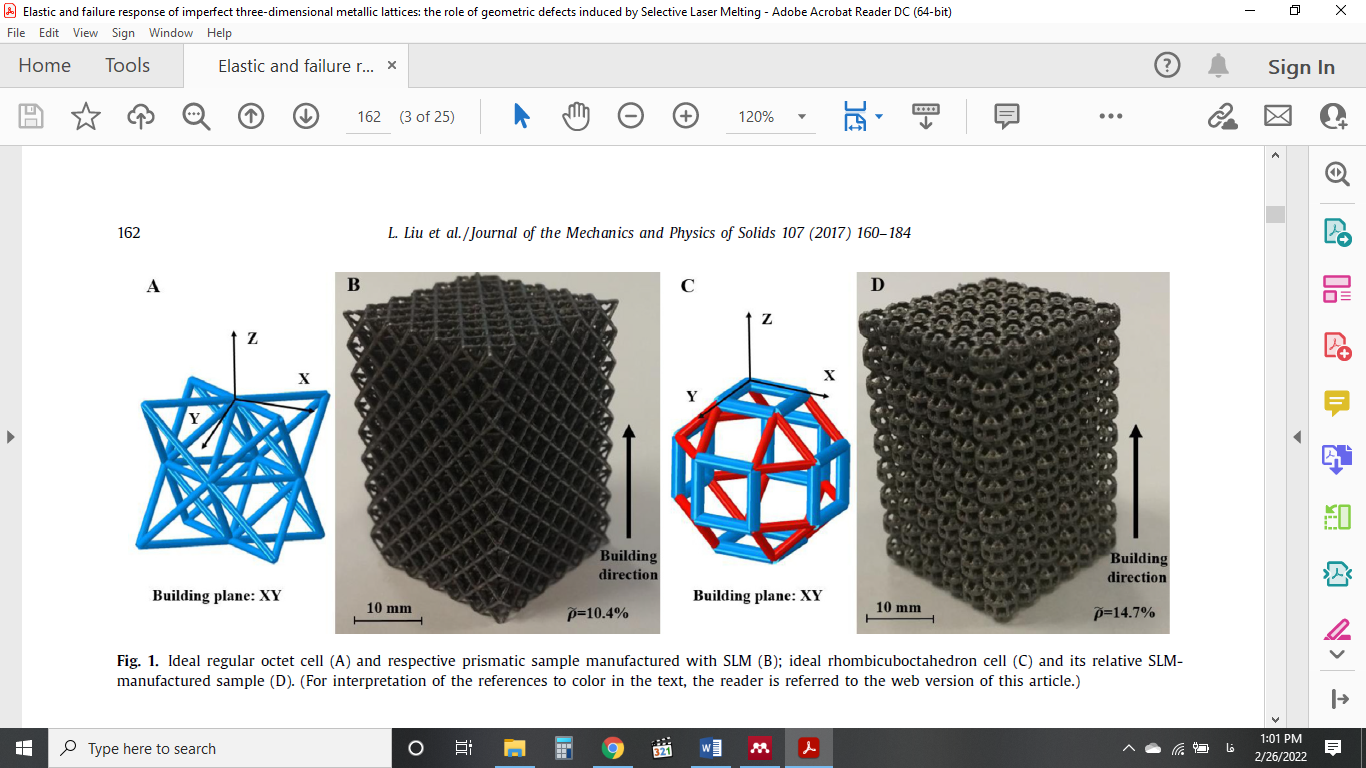 | Ref. [64] |  | Ref. [59] |
| 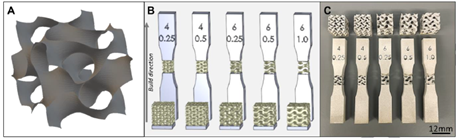 | Ref. [61] | 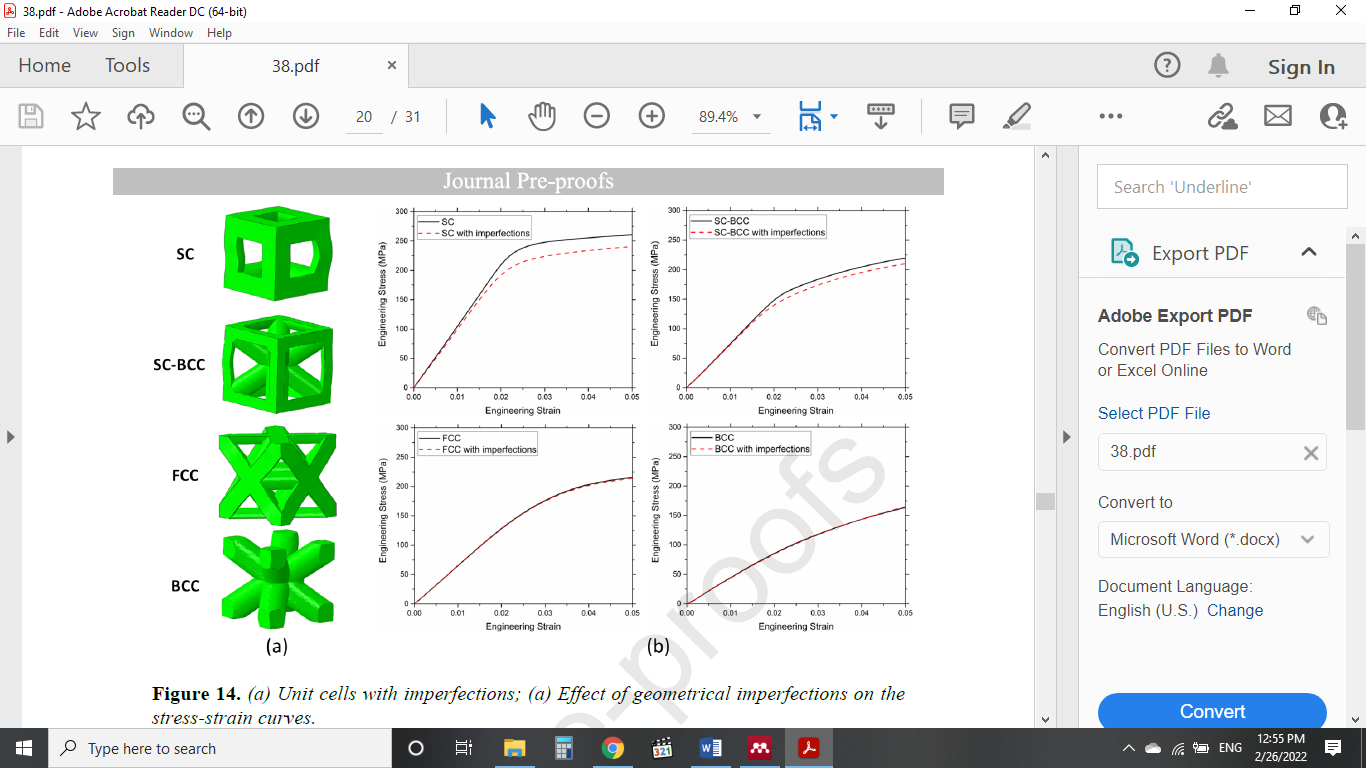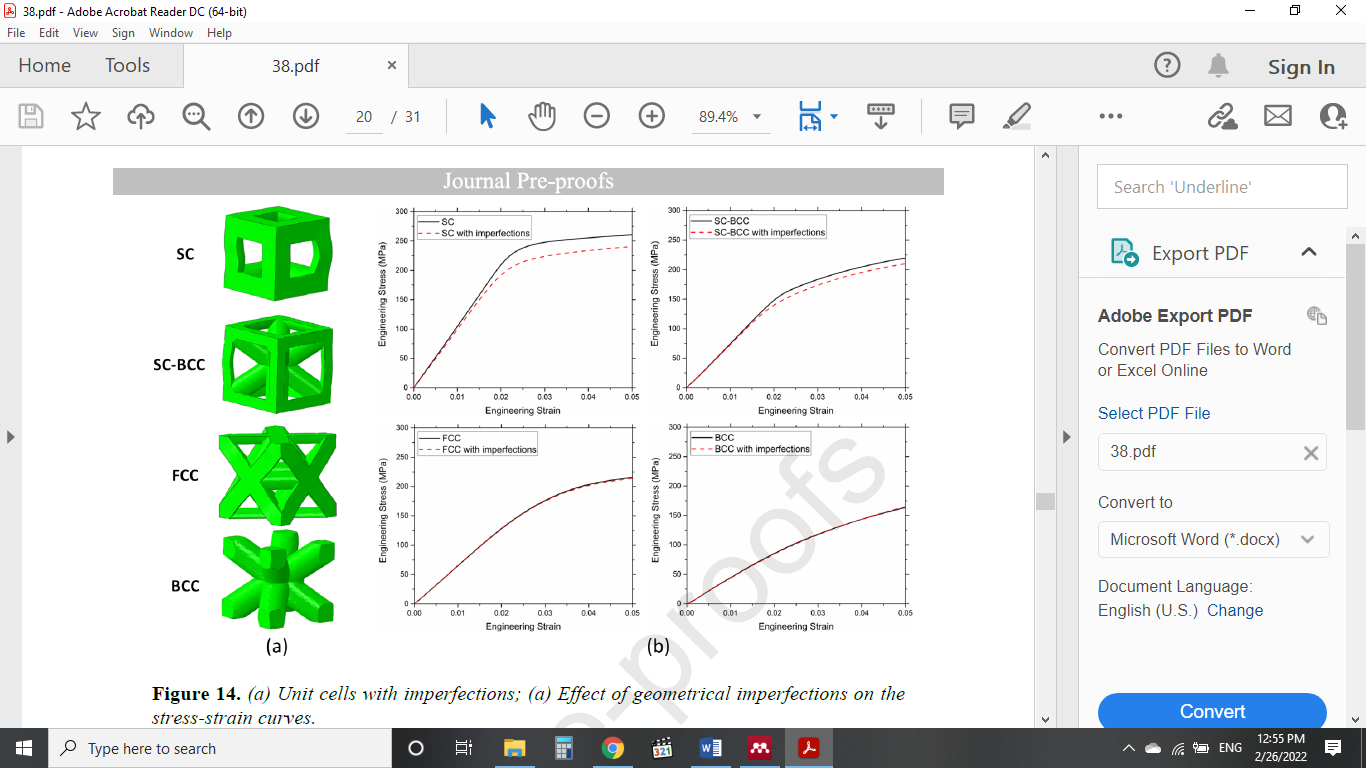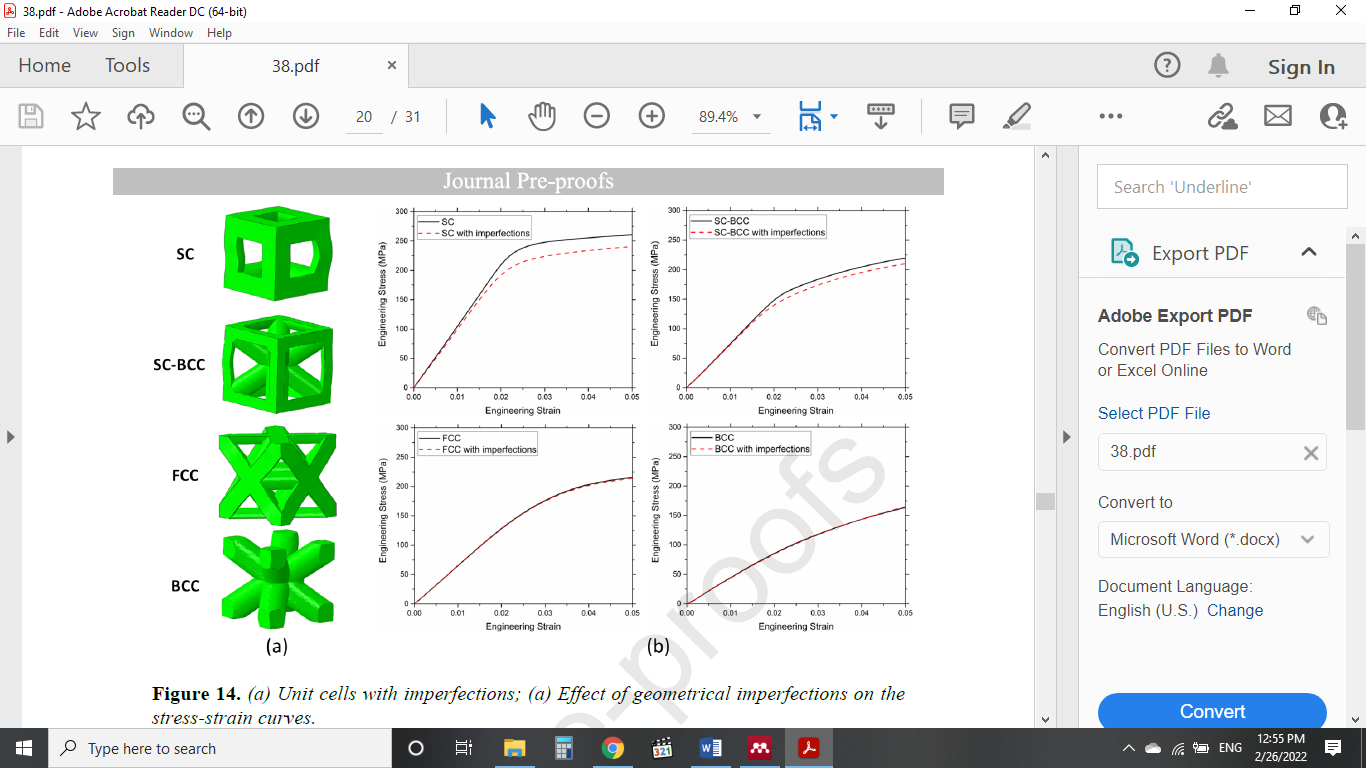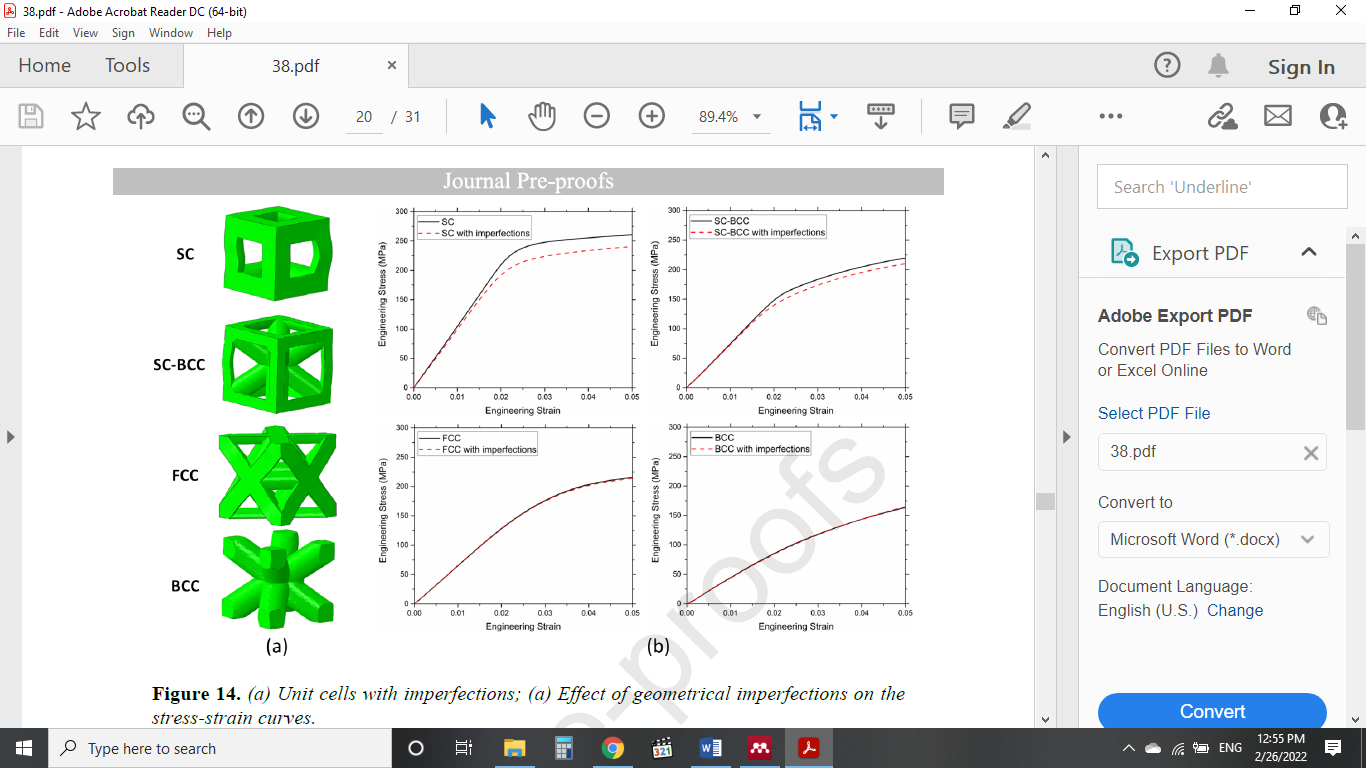 | Ref. [60] |
|  | Ref. [65] |  | Ref. [62] |
|  | Ref. [67] |  | Ref. [66] |

Figure B1: The geometry of different cells in Table B1


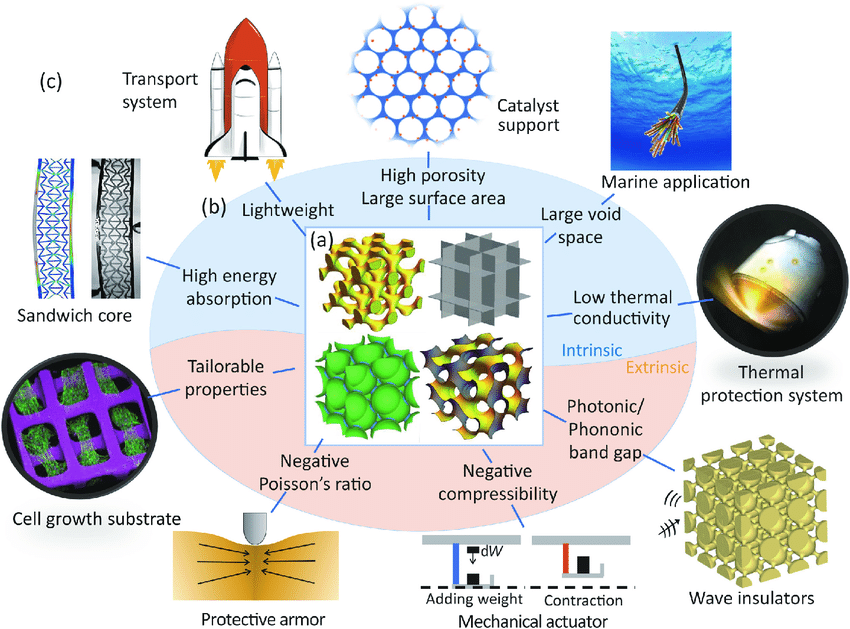


Figure B2: The application for several types of above metamaterial cells
