# Supplementary material for: Topology optimization on metamaterial cells for replacement possibility in non-pneumatic tire and the capability of 3D-printing
Source: PLoS One. 2023 Oct 13;18(10):e0290345. doi: 10.1371/journal.pone.0290345 (PMC10575546; doi:10.1371/journal.pone.0290345)
Supplement: S3 File — (DOCX) [file pone.0290345.s004.docx]

**S3 File: Tensile Testing**

In order to find the properties of the materials used in the ABAQUS software, the tensile tests were performed. Figure C1 shows the map of the standard samples with the dimensions and specifications. Figures C2 and C3 illustrate the specimens before and after tensile testing. The fracture surface of these samples has been checked with a field-emission scanning electron microscopy (FE-SEM).


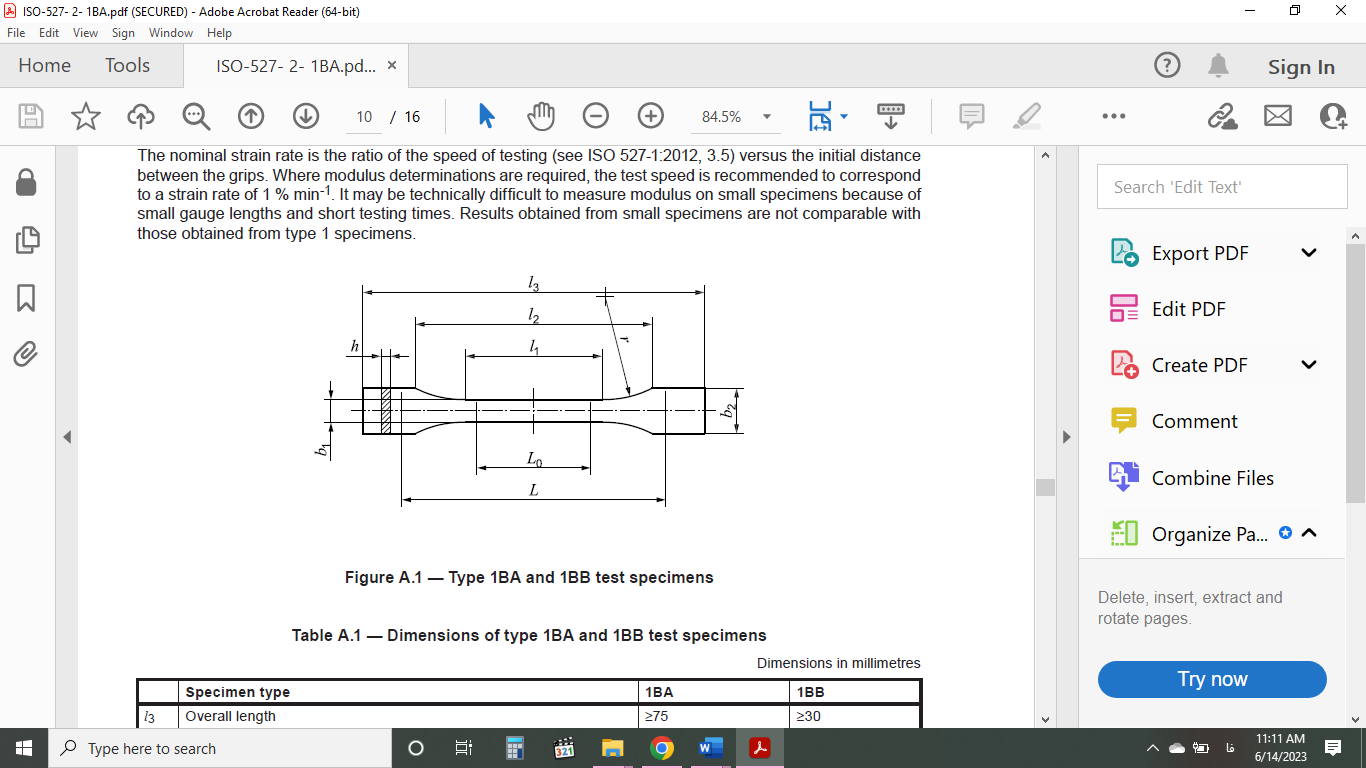


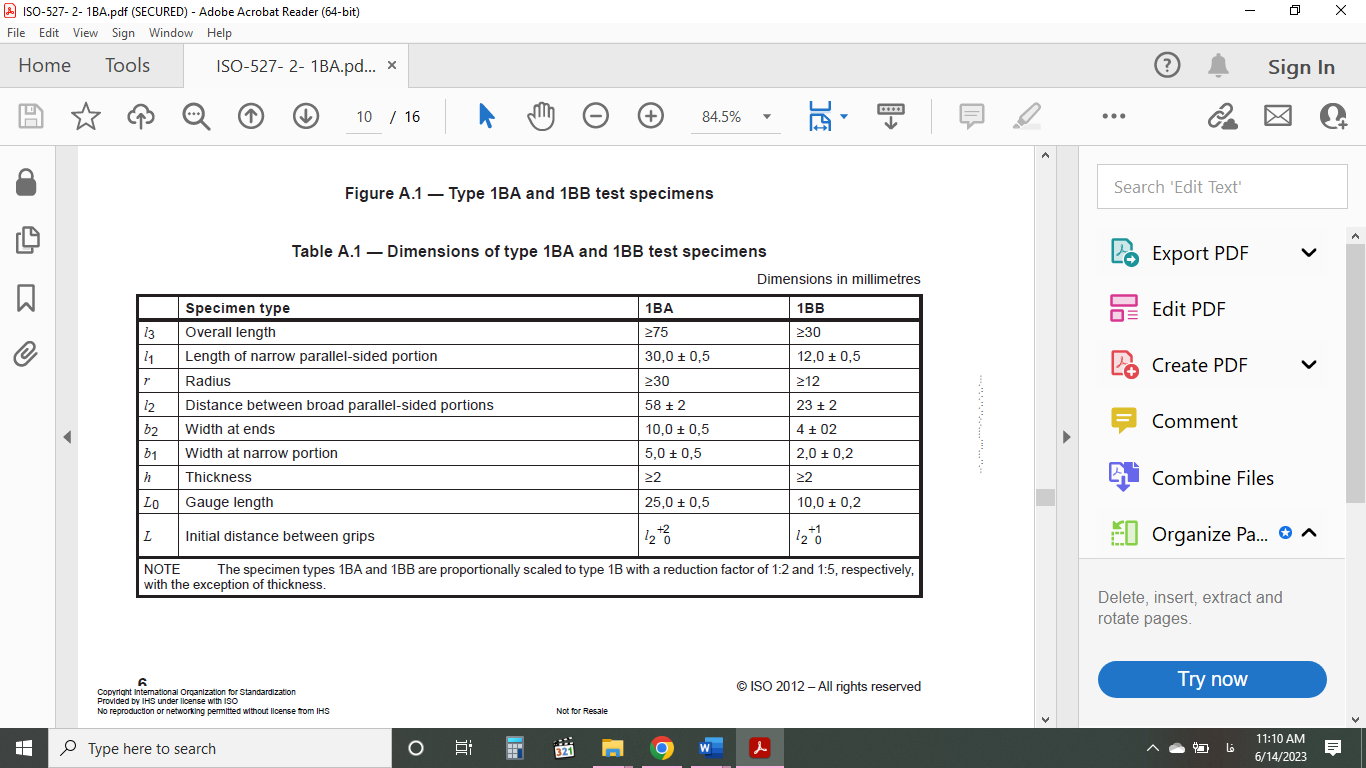


Figure C1: The map of the standard samples and the dimension (in millimeters)


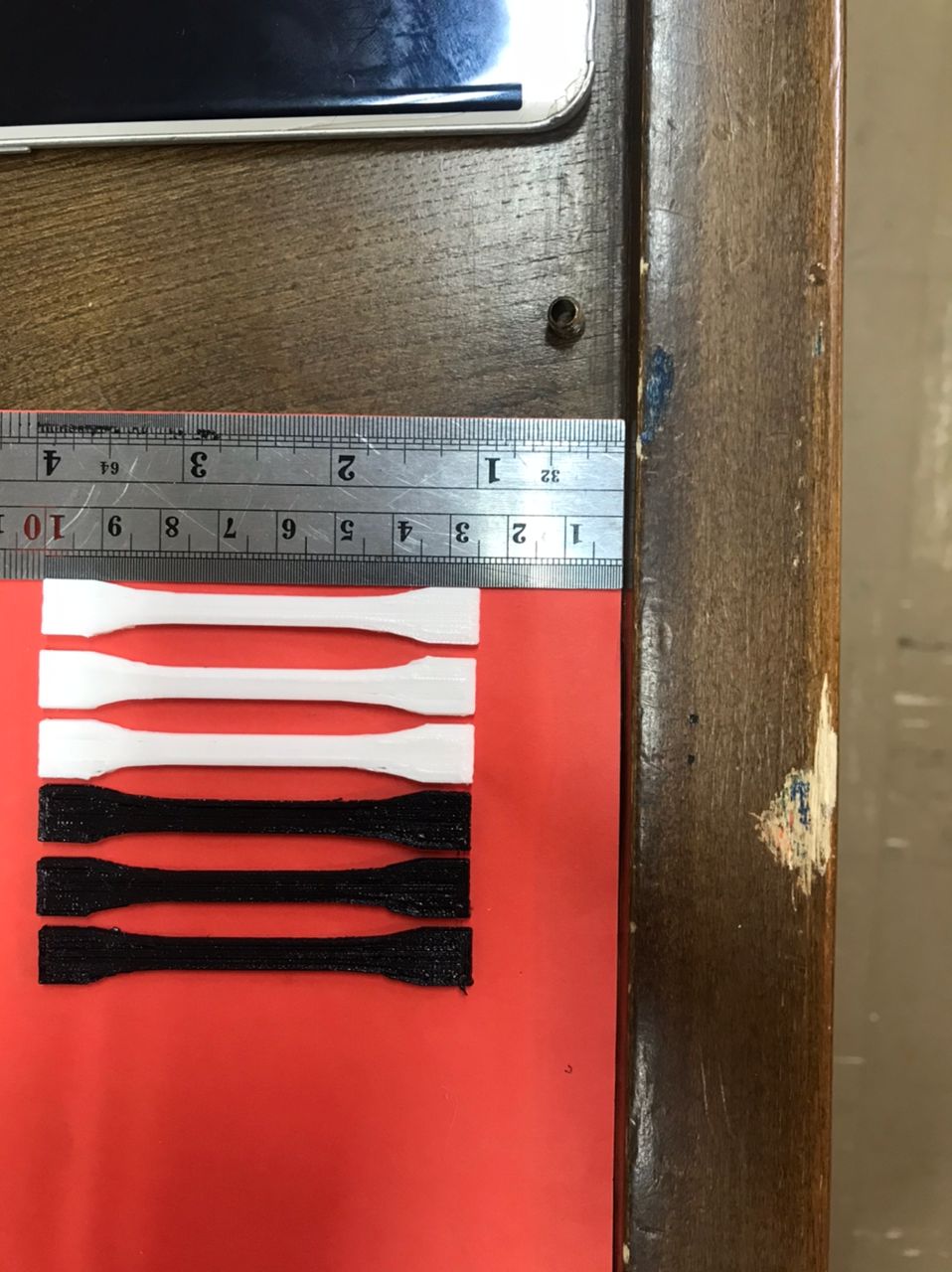


Figure C2: Tensile test samples fabricated by a 3D printer


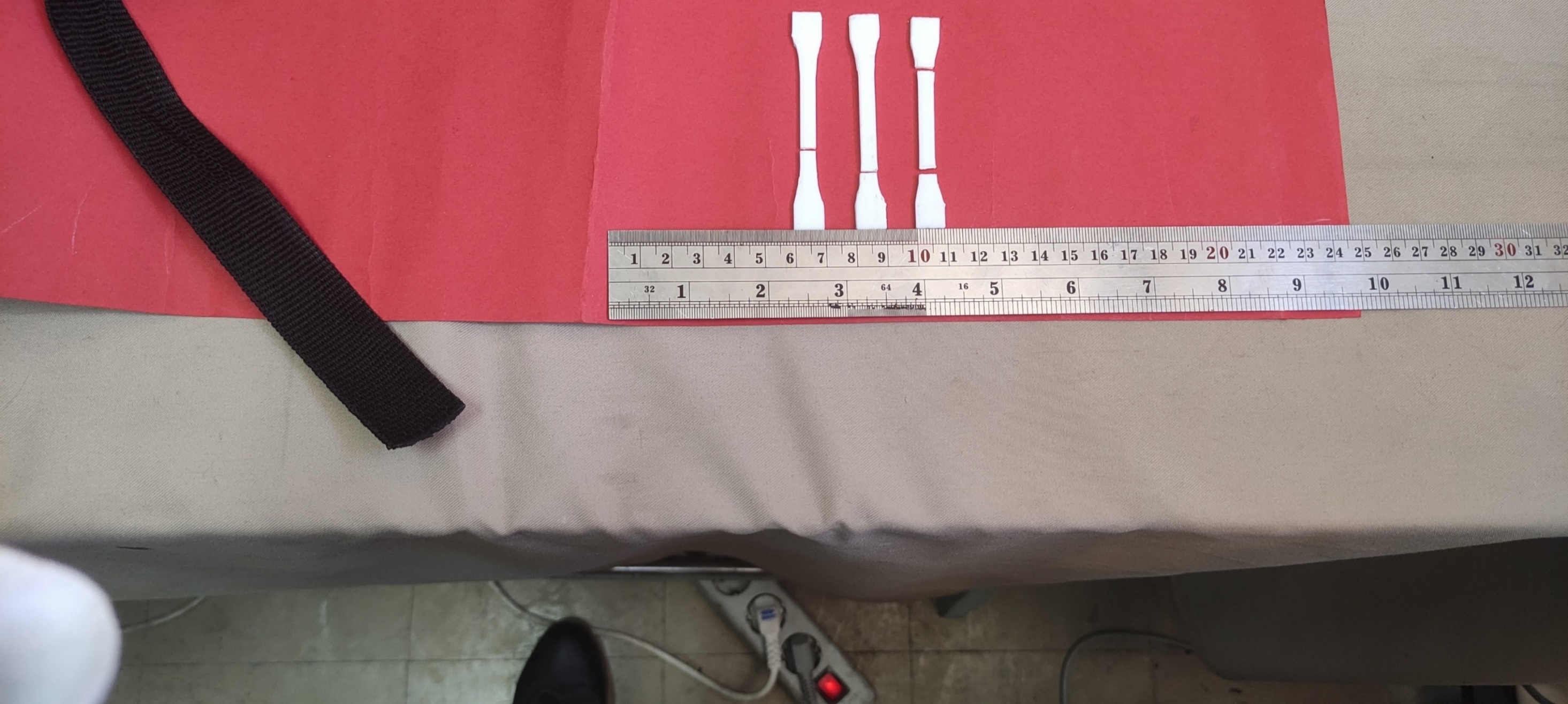


Figure C3: The failed samples made of PLA after tensile testing
