# Supplementary material for: Topology optimization on metamaterial cells for replacement possibility in non-pneumatic tire and the capability of 3D-printing
Source: PLoS One. 2023 Oct 13;18(10):e0290345. doi: 10.1371/journal.pone.0290345 (PMC10575546; doi:10.1371/journal.pone.0290345)
Supplement: S4 File — (DOCX) [file pone.0290345.s005.docx]

**S4 File: Topology optimization**

Topology optimization is essentially creating macroscopic changes between one or more materials, one of which can be considered a hole. In other words, in topology optimization, we are looking for the distribution of material or materials in the basic geometry in such a way that the design objective is optimized with limitations. For this purpose, the basic geometry with a high number of discrete elements and the element density of the material is considered a design variable. In the optimization operation, the properties of the elements are calculated and finally, some elements are removed or the properties of the materials are taken. The final output of this mode of optimization in a discrete mode is called checker design, as shown in Figure D1 (a).

(a) (b)

|  |
| --- |

Figure D1: The topology optimization including (a) discrete design and (b) continuous design (Note: White color is Material 1 and black is Material 2.) [85]

In this checkered design, the white elements are related to one material and the black elements are related to another material, and in a special case, each of these materials can be void. Checkered design or black and white is dependent on the design variable, which can have a value of 0 or 1 and change the characteristics of the material in each element. In this case, the optimization problem is defined according to Equation (D1) [85].

| (D1) | $Min:Ф (C (\rho))$  $s.t.: \sum_{e=1}^{N} \frac{v_{e}\rho_{e}}{v_{0}}\leq f$  $:g_{i}\left( C \left( \rho\right) \right)˂g_{i}^{*} , i = 1, \ldots, M$  $:\rho_{e}=\left\{ \begin{aligned} 0 \left( material 1 \right) \\ 1 \left( material 2 \right) \end{aligned} \right. , e=1, \ldots, N$  $:K(\rho)U_{i}= F_{i} , i = 1, \ldots, m$ |
| --- | --- |

In this regard, minimizing *Ф* is the goal of the problem, which includes *C*, that is a function of the Design variable and is calculated using Equation (D2). *C* is an interpolation function that determines the properties of each element. These properties can express different physical values such as material stiffness, cost, etc. $v_{e}$is the element volume

($v_{0}$) is the total volume in the unit cell, *f* is the volume fraction limit, $g_{i}\left( C \left( \rho\right) \right)$is the problem limit and $g_{i}^{*}$is the limit. The relationship of *K(ρ)U_i_ = F_i_* also represents the finite element system for different loading cases.

| (D2) | $C\left( \rho\right)=\left( 1-\rho_{e} \right) C^{1}+ \rho_{e}C^{2}$ |
| --- | --- |

In Equation (2), $C^{2}$ and $C^{1}$are the material tensors of the two components. If $\rho_{e}$=0, the properties of the element are considered according to the first material, and if $\rho_{e}$=1, the properties of the element are considered according to the second material, and thus a checkerboard design is obtained. However, this design brings problems, including the creation of very delicate and unmanufacturable geometric designs and details. To solve this problem, geometric constraints can be used. However, replacing integer variables with continuous variables is a suitable solution. In fact, by allowing the variables of the design to take any value (between 0 and 1), the discrete optimization problem becomes a continuous problem according to Figure D1 (b), which is called a gray design. In fact, in discrete optimization problems, the optimal connection of elements is checked, and in continuous problems, the optimal distribution of materials in a domain is determined [82]. In the continuous state, the optimization problem is defined according to Equation (D3) [85].

| (D3) | $Min:Ф (C (\rho))$  $s.t.: \sum_{e=1}^{N} \frac{v_{e}\rho_{e}}{v_{0}}\leq f$  $:g_{i}\left( C \left( \rho\right) \right)˂g_{i}^{*} , i = 1, \ldots, M$  $:{0 \leq\rho}_{e}\leq1 , e=1, \ldots, N$  $:K(\rho)U_{i}= F_{i} , i = 1, \ldots, m$ |
| --- | --- |

In this method, there is a possibility that the answers will end up in the middle values of the design variables. Therefore, to avoid this problem, different penalty plans are used. For example, it is possible to use the Solid Isotropic Material with Penalization (SIMP) approach, where the design variable reaches the power of the penalty factor (*p*>1) and directs the solution towards the discrete values 0 and 1. In fact, despite continuous variables, it performs discrete solutions. In this case, the interpolator function is defined as Equation (D4).

| (D4) | $C\left( \rho\right)=\left( 1-\rho_{e} \right)^{p}C^{1}+{\rho_{e}}^{p}C^{2}$ |
| --- | --- |

If the optimization of only one material is considered, the above relation can be replaced with Equation (D5) [83].

| (D5) | $C(\rho) ={\rho_{e}}^{p}C^{1}$ |
| --- | --- |

This relationship is used when one of the two materials in question is void.

In recent years, another interpolator function according to Equation (D6) is used, which is called the modified SIMP method. In this method, the elastic modulus of the cavity is not considered zero to avoid the singularity of the stiffness matrix. One of the advantages of this method is that the minimum value of the elastic modulus of the material and the penalty number is not dependent on each other. In this regard, $c_{min}$ is the elastic modulus of the cavity [84].

| (D6) | ${{c\left( \rho\right)=c}_{min}+\rho}^{p}(c_{0}-c_{min})$ |
| --- | --- |

If the interpolator function C is related to the stiffness of the material, it depends on the elastic modulus and Poisson ratio of the material. In the case that Poisson ratio is independent of the material density and this tensor is supposed to correspond to a composite material made of empty space and the given material with real density, the bulk modulus (*K*) and the shear modulus (*μ*) of the tensor (*C*) should cover the Hashin-Shtrikman range.

For two-phase materials, one phase of which is void. This range is shown in Equation (D7) [83].

| (D7) | $0 \leq K \leq\frac{\rho K^{0}\mu^{0}}{\left( 1-\rho\right)K^{0}+\mu^{0}}$  $0 \leq\mu\leq\frac{\rho K^{0}\mu^{0}}{\left( 1-\rho\right)\left( K^{0}+2\mu^{0} \right)+ K^{0}}$ |
| --- | --- |

In this regard, $K^{0}$and $\mu^{0}$are the bulk modulus and shear modulus of the material in question. In this case, Equation (D8) is calculated for the elastic modulus.

| (D8) | $0 \leq E \leq\frac{\rho E^{0}}{3-2\rho}$ |
| --- | --- |

According to Equation (D5), it can be written:

| (D9) | $0 \leq\rho^{P}E^{0}\leq\frac{\rho E^{0}}{3-2\rho}$ |
| --- | --- |

Equation (D9) is true if and only if *p* is greater than 3. However, in the SIMP model, the Poisson ratio is assumed to be independent of density. In this case, according to the definitions of elastic modulus and shear modulus shown in Equation (D10), the Hashin-Shtrikman range is rewritten as Equation (D11):

| (D10) | $K=\frac{E}{2\left( 1-\nu\right)}$  $\mu=\frac{E}{2\left( 1+\nu\right)}$ |
| --- | --- |
| (D11) | $0 \leq\frac{\rho^{P}E^{0}}{2\left( 1-\nu\right)} \leq\frac{\rho E^{0}}{4-2\left( 1+\nu\right)\rho}$  $0 \leq\frac{\rho^{P}E^{0}}{2\left( 1+\nu\right)} \leq\frac{\rho E^{0}}{2\left( 1-\rho\right)\left( 3-\nu)+2(1+\nu) \right)}$ |

From the above relationship, a condition for calculating the value of *p* according to Equation (D12) is obtained.

| (D12) | $p \geq\max( \frac{2}{1-\nu}, \frac{4}{1+\nu})$ |
| --- | --- |

For example, the *p* factor for materials with different Poisson ratios is shown in Equation (D13).

| (D13) | $if \nu=\frac{1}{3} , p=3$  $if \nu=\frac{1}{2} , p=4$ |
| --- | --- |

In the 3D case, the Hashin-Shtrikman range leads to Equation (D14):

| (D14) | $p\geq\max(15\frac{1-\nu}{7-5\nu} ,\frac{3}{2} \frac{1-\nu}{1-2\nu})$ |
| --- | --- |

In this case, for example, the number p is calculated as follows(D15):

| (D15) | $if \nu=\frac{1}{3} , p\geq3$  $if \nu=\frac{1}{5} , p\geq2$ |
| --- | --- |

In general, the larger the *p* number, the better the average densities are removed. But the solution time increases [86]. Considering that the Poisson ratio is considered 1/3 for the materials used in this research, therefore, a penalty number of 3 is also considered in the analyses carried out in this research.

In the optimization process, sensitivity analysis should be performed. Sensitivity analysis shows which design parameters and variables have the greatest effect on the result. If a variable changes slightly, yet changes the result to a large extent, that variable is of greater importance. But if the variable includes many changes and the result has minimal change, then the design variable is less important. Therefore, the sensitivity of a parameter is calculated based on the derivative of that parameter according to the design variable. The importance of each design variable is effective in the optimization process, as it would be utilized to obtain how uncertain the result is according to various parameters. The high importance of a parameter makes the solution easily changeable depending on the design inputs.
