# Supplementary material for: Topology optimization on metamaterial cells for replacement possibility in non-pneumatic tire and the capability of 3D-printing
Source: PLoS One. 2023 Oct 13;18(10):e0290345. doi: 10.1371/journal.pone.0290345 (PMC10575546; doi:10.1371/journal.pone.0290345)
Supplement: S5 File — (DOCX) [file pone.0290345.s006.docx]

**S5 File: Devices**

In this section, the images of the used devices are shown. Figure E1 shows the image of the 3D printer for polymers, Figure E2 indicates the rotational bending fatigue testing device, and finally, Figure E3 depicts the compressive testing device.


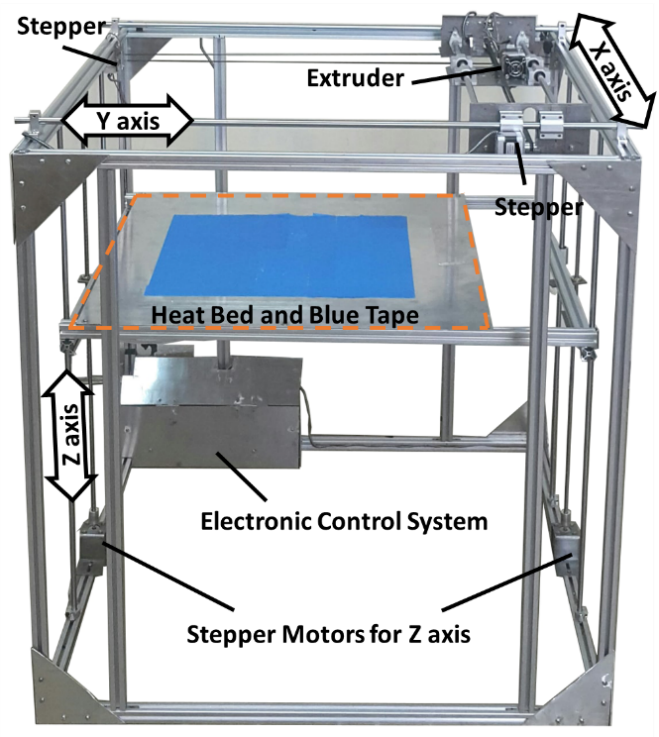


Figure E1: 3D-Printing device for polymers


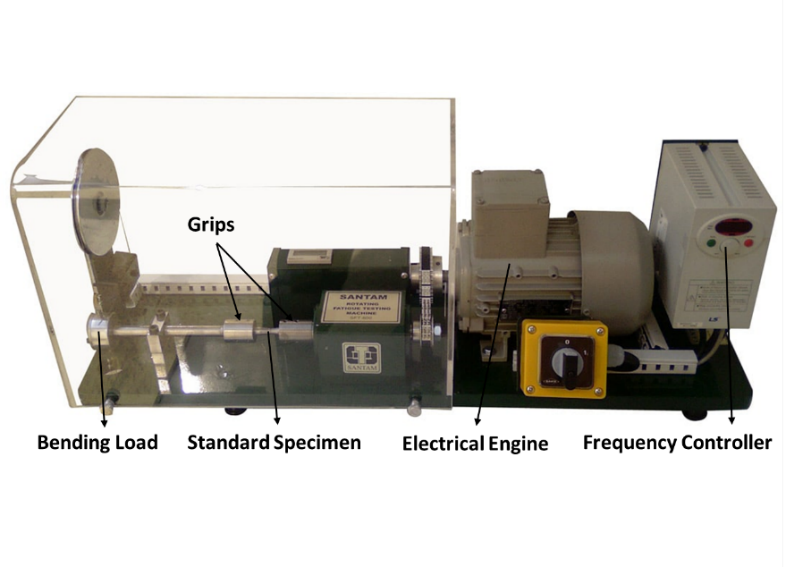


Figure E2: The rotational bending fatigue testing machine


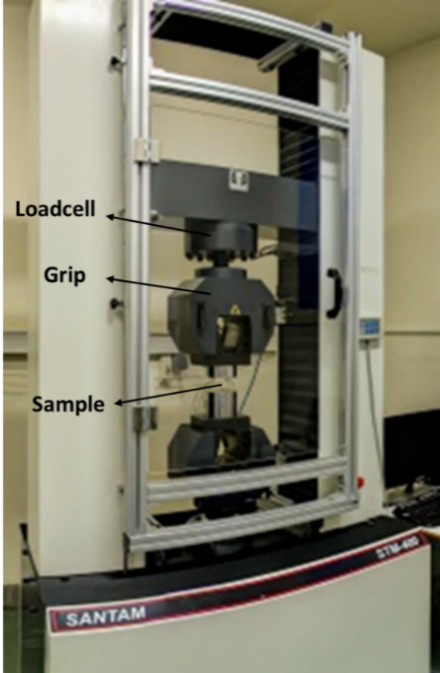


Figure E3: The compressive testing machine
