# Supplementary material for: Topology optimization on metamaterial cells for replacement possibility in non-pneumatic tire and the capability of 3D-printing
Source: PLoS One. 2023 Oct 13;18(10):e0290345. doi: 10.1371/journal.pone.0290345 (PMC10575546; doi:10.1371/journal.pone.0290345)
Supplement: S6 File — (DOCX) [file pone.0290345.s007.docx]

**S6 File: Arrangement of cells in NPTs**

As depicted in Figure 3, the optimized cell can be placed in the tire in a scaled form. Figure F1 shows the cell placement in the tire. When the force is applied to this NPT, the adjacent cell structures will also have a strong effect on each other, which can affect the structural stress change.


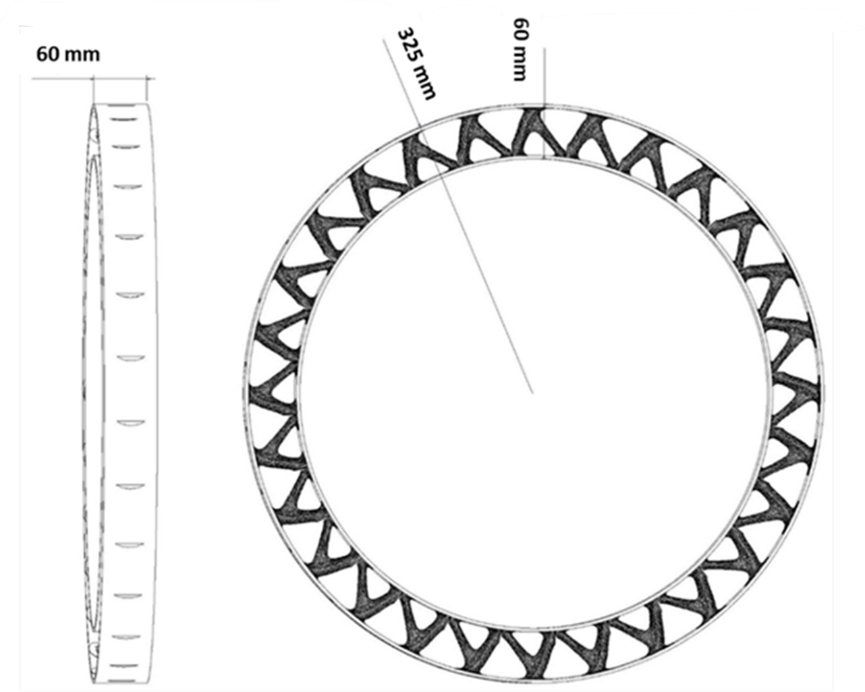


Figure F1: The arrangement of topology-optimized cells in the NPT
