# Supplementary material for: Topology optimization on metamaterial cells for replacement possibility in non-pneumatic tire and the capability of 3D-printing
Source: PLoS One. 2023 Oct 13;18(10):e0290345. doi: 10.1371/journal.pone.0290345 (PMC10575546; doi:10.1371/journal.pone.0290345)
Supplement: Attachment — Submitted filename: Revision Paper PONE-D-23-07715.pdf [file pone.0290345.s008.pdf]

Manuscript Draft: Topology Optimization on Metamaterial Cells for Replacement Possibility in Non-Pneumatic Tire and the Capability of 3D-printing with Polylactic Acid Filaments

PONE-D-23-07715

General Comments:

- Major English revision is needed. Also, some parts of the document include repetitions which could be consolidated to obtain a more concise and clearer document. Some of the analyses of the results are too extensive and confusing due to the high number of different nomenclature used.

- Can the authors comment on the adequacy of PLA material in the production of car's tires?

- The authors mention very often that the tire is subject to bending and compressive loads, but there is a chapter explaining the tensile tests performed to obtain mechanical properties of the material used to be provided to the optimization software. Could the authors elaborate on the adequacy of this choice?

- The authors aim at minimizing compliance / strain energy, meaning they are aiming at the stiffest cell possible that respects the optimization requirements. Can the authors elaborate if for a tire, which should also fulfil a damping behaviour and is usually not very stiff (air pressure), the optimization function chosen is the most adequate?

- In the document, there is a discussion regarding printing orientation. Can the authors comment why is this necessary? The question would only be relevant if the final product could be printed horizontally and vertically using FFF, but wouldn't a vertically printed wheel be unbalance in the end, and wouldn't the unit cells that compose its structure be radially varying, making the "optimization of cells considering printing orientation" not adequate? Or when the authors mention printing orientation of the unit cells it should be understood as the orientation of the cells within an horizontally printed wheel (if so, what would be the circular shape of the optimization results, e.g. figure 12)?

In Introduction:

Page 2

The sentence "In general, cellular structures can be divided into four categories of foams, lattice, and Triply Periodic Minimal Surfaces (TPMS), which include solid-based TPMs and sheet-based TPMs [3]" seems incorrect since four categories are named but only three defined. Please, clarify.

In the sentence "PTs in general have higher stability and longevity compared to the traditional tires and their fabrication via 3Dprinting will result in energy and cost saving [8-9]",ccan the authors elaborate on how the stability and longevity of NPTs is greater than traditional tires, and explain if there is no alternative to 3D-printing NPTs? Maybe consider rephrasing to "could result in" or similar in intent.

The acronym "MEW" has not been defined.

When displaying the wheel design by Zhang et al. [33], consider using stiffness instead of hardness. It should be more adequate if the analysis was concerned with the deformation of the structure rather than with mostly a surface deformation phenomenon. Please comment.

Page 3

The sentences “Ju et al. [5] investigated hexagonal honeycomb cells in NPTs. Two types of hexagonal honeycomb are designed: same load carrying capacity and same cell wall thickness.” is not clear. Also concerning what follows “The results show that cells with a very positive angle under the same vertical bearing capacity have low local stresses and low mass” it is not clear what are positive or negative angles. Could the authors maybe clarify what is meant with cells being characterized by low mass?

Authors have yet to explain fundamental concepts to say, “Using the overhang control in connection with 3D printing of non-pneumatic tires, so that there was no need for the support structure, and it provided better bending and compressive properties”. Please consider rephrasing to a more general and comprehensive bullet at this stage of the article.

Page 4: Where is table 1? Starts with table 2...

Page 5:

Authors state "According to this figure, only cells that are at least 3 times the size of the selected cell can successfully be fabricated via FDM 3D printing" whish might be true for the manufacturing parameters and conditions used in this study, but is not necessarily in the case of other works.

The suggestion is to make that clear.

Page 7

It is not clear what "L, and L0" in Figure 5 represent. Please justify.

Page 9

Even though the condition of the overhang angle is a known phenomenon in FDM, it is not clear what Figure 7 wants to represent...Please clarify.

Page 10

It is not clear what is the meaning of the stress values shown in Figure 8, ... Please clarify.

Page 11

If the tire alternates its stress state between bending / compression, and the ratio of -1 used in the fatigue tests. The effect of average stress was considered? A comment would be apreciated.

Page 14

Please verify the units mentioned in "The results of the optimization of the cylindrical unit cell with a diameter and height of 1 m"

Page 17

Please clarify the analysis of the results as they are not easily understood in their current form (1st paragraph).

Page 22

In section 3.2 authors state that "As mentioned in the third chapter, the sample is closed by 6 screws at a distance of one centimetre from the beginning and the end of the sample." But there is no other place in the document where screws are mentioned. Moreover, can the authors clarify the meaning of "Therefore, this part of the sample is considered for closing fixed screws."

Page 26

Can the authors clarify what is the meaning of a “0.7 lifetime” in the sentence “According to the results, in general, it can be said that the normal lifetime of TO and SC samples is the same on average and is around 0.7.”

Images of Table 21 are not with a proper resolution.

The sentence “These samples are designed for use in car tires. Manufacturers are always looking for lower weight in the car. Because the lighter car weight brings benefits such as reducing fuel consumption and emissions. Therefore, if the designer is looking for lightweight design of non-pneumatic tires, he can use this type of cell.”

This is a clear example of a paragraph that need to be English revised. Also, this sentence should be relocated, potentially to the final conclusions or introduction, since similar content in these sections of the document already exist.

Page 31

Figure 23 could be improved. “Defect caused by manufacturing method” is not a conclusive cause of defects. It is not clear what was the printing orientation of the specimens. Please add a comment, and characterize the different defects.

Page 33

Could the authors verify the first bullet according with a previous comment? Also, the same bullet says 4.2 mm, is this the same conclusion of figure 1?
