# Supplementary material for: Topology optimization on metamaterial cells for replacement possibility in non-pneumatic tire and the capability of 3D-printing
Source: PLoS One. 2023 Oct 13;18(10):e0290345. doi: 10.1371/journal.pone.0290345 (PMC10575546; doi:10.1371/journal.pone.0290345)
Supplement: Attachment — Submitted filename: Answers to comments2.docx [file pone.0290345.s009.docx]

**Dear Editor-in-Chief,**

The article, entitled “*Topology Optimization on Metamaterial Cells for Replacement Possibility in Non-Pneumatic Tire and the Capability of 3D-printing with Polylactic Acid Filaments*”, is thankfully reviewed by your nice journal. First of all, thank you for giving us the chance of submitting a revision. Then also, we should thank the respected reviewers for their nice comments. All mentioned notes were addressed in the revised text and changes were also highlighted in yellow-colored sentences. Moreover, answers to all comments could be found in the following paragraphs.

Regards,

**M. Azadi, PhD.**

**Reviewer #1:**

The paper presents an interesting analysis on the design of structures for the use in non-pneumatic tires.

However, there are comments and questions that need to be addressed.

Answer: First of all, the authors should thank the respected reviewer for the nice comments. We have tried our bests to address all comments in the revised article.

1. P.2, l.2 …” Poisson ratio close to zero [1].”- besides reference 1, the authors should also cite the book <https://doi.org/10.1017/CBO9781139878326>, Lorna J. Gibson and Michael F. Ashby

Answer: Thank you. Your suggestion as a very valuable book has been introduced. Therefore, reference 1 is changed to the following literature,

*[1] L.J. Gibson and M.F. Ashby, "Cellular Solids Structure and Properties" Cambridge University Press, 2014.*

2. P.2, which are extensively researched and presented in Appendix A. Why did the authors choose to separate this review from the introduction? The text from Appendix A could be compressed and inserted here. In fact, all the introduction should be shortened.

Answer: All reviewed articles are briefly mentioned in the introduction. Appendix A has been written for more information. As you have also mentioned the introduction is lengthy, if we add this appendix, it will be more. Therefore, we decide to separate this part as an appendix. If necessary, we could change it again, in the next revision. As another note, the mentioned literature in Appendix A is based on products in industrial companies, which are not scientific works, and therefore, they were separated.

3. P.3 “Mazur [9] has compared a non-pneumatic tire with pneumatic tires. It has been concluded that the rolling resistance force of non-pneumatic tires is different from pneumatic tires” how different? Smaller or larger?

Answer: Concerning the reviewer's opinion, this text is changed to the following text,

*It was concluded that experimental results did not contradict the claims of manufacturers that NPT had higher damping properties while having lower rolling resistance.*

The details of values are not important in this case study, which is only a literature review.

4. P.3 “Two types of hexagonal honeycomb are designed: same load carrying capacity and same cell wall thickness.” Please explain the difference between the cell structure.

Answer: Thanks for the nice opinion. In order to address the respected reviewer’s comment, the following sentences were added to the revised article.

*Ju et al. [5] introduced a NPT with a compliant cellular solid spoke component. In that work, hexagonal honeycomb spokes for a high fatigue resistance design were investigated by seeking compliant hexagonal structures that had low local stresses under macroscopic uniaxial loading. Using the honeycomb mechanics, two cases of hexagonal honeycombs were designed, (1) the same load-carrying capacity and (2) the same cell wall thickness. The results demonstrated that the hexagonal honeycombs with a highly positive cell angle had low local stresses and low mass under the same vertical load carrying capability.*

5. P.3, “Finding a cell with a resistance to compressive forces and bending cyclic loads (fatigue).” Compared to what?

Answer: With respect to the reviewer’s opinion, this text is changed to the following sentence,

*Finding a cell with a resistance to compressive forces and bending cyclic loads (fatigue), compared to other cells and solid tires.*

6. P.4, 2.1) Patterning of cells: please add that the cells of Appendix A are used in several applications.

Answer: With respect to the reviewer’s comment, the following figure is added in the appendix, as follow,

*In addition, Figure B1 shows the application for these cells.*

Figure B1: The application for several types of metamaterial cells

7. P4. “Table 1 in appendix B”, it should be Table B1.

Answer: Thanks for the nice opinion of the respected reviewer. It was corrected in the whole revised text.

8. Replace all over the text FDM by FFF (fused filament fabrication), as FDM is a trade mark.

Answer: With respect to the reviewer’s opinion, all FDM words were changed to FFF.

9. P.7 “The main material which used is poly lactic acid (PLA).”, please explain the use of a rigid material and not the use of for example a rubber?

Answer: That is a good question. With respect to the opinion of the respected reviewer, the reason for this choice was mentioned in the manuscript as the following sentences,

*It should be noted that in linear finite element modeling, the change in material properties in the elastic region does not change the von-Mises stress distribution in the structure. Therefore, there is no change in the final optimization response. In other words, the stress* *is dependent on the geometry of the* *part and independent of the material properties [89].*

Therefore, there is no change in the elastic linear behavior when the material properties change. Finally, using PLA or TPU in the elastic behavior has no effect on the results. However, this nice opinion to use the rubbery filament such as TPU was published in the previous article by the authors [89]. However, in order to address this nice comment, the following sentences were added to the revised article for further investigations,

*The image of the NPT with the optimized cell for the future research is shown in Appendix F.* *Moreover, in further investigations, the following issues will be considered and studied,*

- *The mechanical properties of this optimized tire including the compressive behavior and the fatigue performance,*
- *Different arrangements between the unit cell under compressive loads,*
- *The behavior of hyper-elastic materials such as TPU (as rubbery structures) in the optimization process, besides considering the damping behavior.*
- *Evaluating the effect of mean stress on the structure performance during fatigue testing.*

Finally, the material type is also removed from the article title to show that the material type was not the focus of this manuscript.

10. P.7” The main material which used is poly lactic acid (PLA).” There is a missing word. There are some typos in the text. Please correct it.

Answer: Thank you for the comment and sorry for mistakes. It was changed to the following sentence,

*The studied material was poly lactic acid (PLA).*

11. Appendix C also should be inserted in the text of section 2.3

Answer: With respect to the opinion, Appendix C (Appendix D in the revised article) is for more information. In addition, in order to address your nice opinion (Comment 16) and to reduce the text length, we used this additional description in the appendix. If necessary, we could move it to the main text, in the next revision.

12. “According to Table 7, the example of row 4 is chosen as the optimal example of the cubic unit cell due to having the lowest weight.” Please explain why it is not row 7.

Answer: With respect to the opinion of the respected reviewer, the reason for this choice was mentioned in the manuscript in the following sentences,

*But* *since the continuity of the cell was lost in the constraint of 20%, other steps of optimization were performed with a constraint greater than this value to calculate the most optimal cell.*

13. Figure 13 – could the authors discuss the defects obtained in the smaller specimen?

Answer: According to the respected reviewer’s comment, the following text was added,

*As shown in this figure, the FFF 3D printer was not capable of producing the smallest scale. This issue was due to the diameter of the nozzle (0.2 mm). If laser-based printers are used, due to the higher accuracy, cells with smaller scales can be produced.*

14. P. 22, explain what do you mean by “As mentioned in the third chapter…? What chapter or what section?

Answer: Thanks for the comment and sorry for this mistake. The text was modified to the following sentence,

*The sample was closed by 6 screws at a distance of 1 cm from the beginning and the end part of the sample. Therefore, this part of the specimen was freeze. In fact, the geometric constraint of freezing was considered for this part of the sample.*

15. P.31 “The image of compressed solid samples is shown in part a of Figure 24 and the image of compressed samples with cellular structure is shown in part b of the same figure.”, there is no part a and part b in the caption of the figure.

Answer: The text was modified to the following sentences,

*The image of the fracture behavior in compressive testing is shown in Figure 18, for both solid and cellular structures.*

16. Overall, the authors should shorten the text. The work probably comes from a PhD thesis and it is too long for a unique paper.

Answer: This issue is correct; however, it is from a MSc thesis. Notably, one part of this work was published as the title of “Multi-Material Metamaterial Topology Optimization to Minimize the Compliance and the Constraint of Weight: Application of Non-Pneumatic Tire Additive-Manufactured with PLA/TPU Polymers” in the Polymers journal (MDPI). This article is the second part of the job. However, according to your nice comment, some paragraphs of the manuscript moved to Appendix C, E, and F, in order to reduce the length. These parts are less important in comparison to other sections.

*The image of the NPT with the optimized cell for the future research is shown in Appendix F.*

*More information about tensile testing is provided in Appendix C.*

*The image of the devices, used in this work, is shown in Appendix E.*

**Reviewer #2:**

1- Major English revision is needed. Also, some parts of the document include repetitions which could be consolidated to obtain a more concise and clearer document. Some of the analyses of the results are too extensive and confusing due to the high number of different nomenclatures used.

Answer: First of all, the authors should thank the respected reviewer for the nice comments. We have tried our bests to address all comments in the revised article.

Then, the whole text was reread and revised to improve English writing. Now, the correctness of the manuscript was confirmed with the Grammarly software, with the score of 90.

Moreover, the abbreviation list was provided before the reference part. In addition, some parts were removed and moved to Appendix C, E, and F, in order to reduce the text.

*The image of the NPT with the optimized cell for the future research is shown in Appendix F.*

*More information about tensile testing is provided in Appendix C.*

*The image of the devices, used in this work, is shown in Appendix E.*

2- Can the authors comment on the adequacy of PLA material in the production of car’s tires?

Answer: That is a good question. With respect to the opinion of the respected reviewer, the reason for this choice was mentioned in the manuscript as the following sentences,

*It should be noted that in linear finite element modeling, the change in material properties in the elastic region does not change the von-Mises stress distribution in the structure. Therefore, there is no change in the final optimization response. In other words, the stress is dependent on the geometry of the part and independent of the material properties [89].*

Therefore, there is no change in the elastic linear behavior when the material properties change. Finally, using PLA or TPU in the elastic behavior has no effect on the results. However, this nice opinion to use the rubbery filament such as TPU was published in the previous article by the authors [89]. However, in order to address this nice comment, the following sentences were added to the revised article for further investigations,

*The image of the NPT with the optimized cell for the future research is shown in Appendix F. Moreover, in further investigations, the following issues will be considered and studied,*

- *The mechanical properties of this optimized tire including the compressive behavior and the fatigue performance,*
- *Different arrangements between the unit cell under compressive loads,*
- *The behavior of hyper-elastic materials such as TPU (as rubbery structures) in the optimization process, besides considering the damping behavior.*
- *Evaluating the effect of mean stress on the structure performance during fatigue testing.*

Finally, the material type is also removed from the article title to show that the material type was not the focus of this manuscript.

3- The authors mention very often that the tire is subject to bending and compressive loads, but there is a chapter explaining the tensile tests performed to obtain mechanical properties of the material used to be provided to the optimization software. Could the authors elaborate on the adequacy of this choice?

Answer: Thanks for the nice comment of the respected reviewer. The tensile test was chosen because of the more critical conditions. In fact, a type of safety factor was considered. the reason for this choice was mentioned in the revised manuscript as the following sentence,

*In this research, the tensile properties of materials, which have a more critical effect on the sample behavior, compared to compressive loads, were used.*

In addition, according to the following figure, the mechanical properties of the PLA material are the same in the elastic region, under tensile and compressive testing.

Figure New: The mechanical properties of PLA under tensile and compressive testing

*[Reference: S. Dezianian, MSc Thesis, Semnan University, Semnan, Iran, 2022]*

4- The authors aim at minimizing compliance/ strain energy, meaning they are aiming at the stiffest cell possible that respects the optimization requirements. Can the authors elaborate if for a tire, which should also fulfil a damping behavior and is usually not very stiff (air pressure), the optimization function chosen is the most adequate?

Answer: This comment is correct. However, considering such as additional function makes the manuscript more complex that also mentioned by the respected reviewer in the first comment. Therefore, the damping issue was not considered in this research. Finally, to address this comment, the following text was added to the revised manuscript,

*In pneumatic tires, the air inside the tire and the tire wall have the capability to resistant the forces on the tire. However, in NPTs, there is no air or tire wall. Therefore, the desired geometry and material must be able to resistant these forces. In addition, in the other articles [89-90], the objective function was considered as a minimum compliance**, compared to this work.*

*[90]*	[I. G. Jang](https://www.tandfonline.com/author/Jang%2C+in+Gwun)*,* [Y. H. Sung](https://www.tandfonline.com/author/Sung%2C+Young+Hwa)*,* [E. J. Yoo](https://www.tandfonline.com/author/Yoo%2C+Eui+Jong)*,* [B. M. Kwak](https://www.tandfonline.com/author/Kwak%2C+Byung+Man)*, “Pattern design of a non-pneumatic tyre for stiffness using topology optimization.”, Engineering Optimization, Vol. 44, pp. 119-131, 2012.*

Moreover, in order to address this issue in a better manner, the future investigations could be considered this comment, as mentioned as follow,

- *The behavior of hyper-elastic materials such as TPU (as rubbery structures) in the optimization process, besides considering the damping behavior.*

5- In the document, there is a discussion regarding printing orientation. Can the authors comment why is this necessary? The question would only be relevant if the final product could be printed horizontally and vertically using FFF, but wouldn't a vertically printed wheel be unbalance in the end, and wouldn't the unit cells that compose its structure be radially varying, making the "optimization of cells considering printing orientation" not adequate? Or when the authors mention printing orientation of the unit cells it should be understood as the orientation of the cells within a horizontally printed wheel (if so, what would be the circular shape of the optimization results, e.g. figure 12)?

Answer: With respect to the opinion of the respected reviewer, the reason for this choice was mentioned in the revised manuscript, as the following sentences,

*The printing orientation affects the mechanical properties of the material. This note is the first issue that is also mentioned in the literature [74–77]. In addition, the printing orientation can affect that either the support is required or not. Therefore, it is very important to check the printing direction. This issue is due to the disadvantages of FFF 3D printers, which needs to have the support during fabrication. In fact, in parts where the angle between the part and the horizontal line is less than a certain value, the supporting structures prevent material excessive deviation from the design geometry [92]. Considering that separating these supporting structures from the main piece is a difficult task and sometimes leads to damage to the surface of the sample, in the optimization process, overhang constraints are used to create angles greater than 45°.*

In Introduction:

Page 2

The sentence “In general, cellular structures can be divided into four categories of foams, lattice, and Triply Periodic Minimal Surfaces (TPMS), which include solid-based TPMs and sheet-based TPMs [3]” seems incorrect since four categories are named but only three defined. Please, clarify.

Answer: Sorry for the mistake. With respect to your opinion, the text was changed to the following sentences.

*In general, cellular structures can be divided into three categories of (1) foams, (2) lattice, and (3) Triply Periodic Minimal Surfaces (TPMS), which include solid-based and sheet-based TPMs [3].*

In the sentence “NPTs in general have higher stability and longevity compared to the traditional tires and their fabrication via 3Dprinting will result in energy and cost saving [8-9]”, can the authors elaborate on how the stability and longevity of NPTs is greater than traditional tires, and explain if there is no alternative to 3D-printing NPTs? Maybe consider rephrasing to “could result in” or similar in intent.

Answer: That job was from the literature. However, in order to address this comment, we have used “could” instead of “will”, as follows,

*NPTs in general have higher stability and longevity compared to the traditional tires and their fabrication via 3Dprinting could result in energy and cost saving [8-9].*

The acronym “MEW” has not been defined.

Answer: Sorry, it is corrected in the revised manuscript, as follows,

*NPTs have a wide range of potential applications among which their use in wheelchairs, space probes, cars, trucks, bicycles, can be pointed out [11]. Various architectures of NPTs have been designed and studied in the past, including Tweel [12, 13, 22, 23, 14–21] and* *Mechanical Elastic Wheel (MEW) [24–31] tires, which are extensively researched and presented in Appendix A.*

When displaying the wheel design by Zhang et al. [33], consider using stiffness instead of hardness. It should be more adequate if the analysis was concerned with the deformation of the structure rather than with mostly a surface deformation phenomenon. Please comment.

Answer: Thanks for your nice accurate review. It is changed to the stiffness.

*Zhang et al. [33] designed a NPT according to the lower body of a kangaroo. By comparing a pneumatic tire with the same size, a radial* *stiffness comparison test was performed to ensure the accuracy of the numerical analysis.*

Page 3

The sentences “Ju et al. [5] investigated hexagonal honeycomb cells in NPTs. Two types of hexagonal honeycomb are designed: same load carrying capacity and same cell wall thickness.” is not clear. Also concerning what follows “The results show that cells with a very positive angle under the same vertical bearing capacity have low local stresses and low mass” it is not clear what are positive or negative angles. Could the authors maybe clarify what is meant with cells being characterized by low mass?

Answer: Thanks for the nice opinion. In order to address the respected reviewer’s comment, the following sentences were added to the revised article.

*Ju et al. [5] introduced a NPT with a compliant cellular solid spoke component. In that work, hexagonal honeycomb spokes for a high fatigue resistance design were investigated by seeking compliant hexagonal structures that had low local stresses under macroscopic uniaxial loading. Using the honeycomb mechanics, two cases of hexagonal honeycombs were designed, (1) the same load-carrying capacity and (2) the same cell wall thickness. The results demonstrated that the hexagonal honeycombs with a highly positive cell angle had low local stresses and low mass under the same vertical load carrying capability.*

Authors have yet to explain fundamental concepts to say, “Using the overhang control in connection with 3D printing of non-pneumatic tires, so that there was no need for the support structure, and it provided better bending and compressive properties”. Please consider rephrasing to a more general and comprehensive bullet at this stage of the article.
Answer: That is correct and therefore, the text was changed to the following one,

- Using the geometry constraint (the overhang control) in the connection with the 3D printing of NPTs, in order to have no supports for the structure.

Page 4

Where is table 1? Starts with table 2...

Answer: Sorry for the mistake. All numbers of tables were checked again and corrected in the revised article.

Page 5

Authors state "According to this figure, only cells that are at least 3 times the size of the selected cell can successfully be fabricated via FDM 3D printing" whish might be true for the manufacturing parameters and conditions used in this study, but is not necessarily in the case of other works. The suggestion is to make that clear.

Answer: The respected reviewer is generally correct. However, the 3D printing capability of the same cell was also reviewed in Table 1. Therefore, the following sentences were modified in the revised article,

*Table 1 also shows the dimensions and number of HC unit cells used in each structure.* *It should be noted that in this table, the discussed cells were produced using laser-based AM, such as Electron Beam Melting (EMB) and Selective Laser Melting (SLM). These processes have higher accuracy and the ability to produce parts with very small dimensions, compared to the FFF technique.*

Table 1: The dimensions and the layout type of the HC unit cell

| Fabrication Process | Cell length (mm) | Cell thickness (mm) | The radius of the cell fillet (mm) | Number of cells | Dimensions of the structure (mm) | Structural geometry | Ref. |
| --- | --- | --- | --- | --- | --- | --- | --- |
| EMB | - | - | - | 5🞨5🞨5 | - | cubic | [54] |
| SLM | 1.4 | 0.3 | - | - | 15×10 | Cylindrical | [56] |
| SLM | 1.24 | 0.26 | 0.13 | - | 13.5×10 | Cylindrical | [52] |
| SLM | 4 | 0.7 | 0.2 | 3🞨2🞨2 | - | cubic | [58] |
| EBM | - | 0.65 | - | - | - | Cylindrical | [63] |
| EBM | 1443 | 0.614 | - | - | 10🞨10🞨20 | cubic | [66] |
| SLM | 15 | 0.26 | 0.13 | - | 13.5× 10 | Cylindrical | [59] |
| SLM | 4 | 1.5 | - | 1  2🞨2🞨2  3🞨3🞨3 | - | cubic | [67] |
| SLM | 4 | 0.67 | 0.6 | 12×11 | - | Cylindrical | [69] |

Page 7

It is not clear what "L, and L0" in Figure 5 represent. Please justify.

Answer: According to the respected reviewer's opinion, Figure C1 is revised (moved from the main text into the appendix).

Figure C1: The map of the standard samples and the dimension (in millimeters)

Page 9

Even though the condition of the overhang angle is a known phenomenon in FDM, it is not clear what Figure 7 wants to represent...Please clarify.

Answer: That is correct and the overhang angle is a known phenomenon in FDM. Therefore, the mentioned figure is removed.

Page 10

It is not clear what is the meaning of the stress values shown in Figure 8, ... Please clarify.

Answer: Thanks for your comment. The following sentence was added to the manuscript,

*Figure 4 shows the difference between the print direction and the overhang control on the final response, including the von-Mises stress and the weight after optimization.*

Page 11

If the tire alternates its stress state between bending / compression, and the ratio of -1 used in the fatigue tests. The effect of average stress was considered? A comment would be appreciated.

Answer: With respect to your nice comment, the mean stress which was considered in this research was zero (R=-1). However, this nice issue could be considered in the future research that will illustrate the effect of mean stress on the structure performance. This issue was mentioned in the revised article before conclusions, as follows,

- *Evaluating the effect of mean stress on the structure performance during fatigue testing.*

Page 14

Please verify the units mentioned in "The results of the optimization of the cylindrical unit cell with a diameter and height of 1 m"

Answer: The mentioned dimension was correct, as also shown in Figure 3. That is a unit cell with the dimension of 1 m. However, with respect to the reviewer’s comment, the mentioned sentence is modified as follows,

*The results of the optimization for the cylindrical unit cell, with a diameter of 1 m and the height of 1 m, …*

Page 17

Please clarify the analysis of the results as they are not easily understood in their current form (1st paragraph).

Answer: With respect to your comment, the text was changed to the following sentence,

*Finally,* *the reason for choosing the optimal cell was as follows,*

- *According to Table 9, the cell with a weight limit of less than 5% (Row No. 7) did not have continuity. Then, it was not selected.*
- *According to Table 11, the stress in the cell with a weight limit of less than 10% (Row No. 6) was higher than the stress in the selected cells (HC, TC, and RD). Then, it was not selected again.*
- *The cell with a weight limit of less than 15% (Row No. 5) had a very small weight change in addition to a high increase in the stress, compared to the cell with a weight limit of less than 20% (Row No. 4). Then, it was also not selected.*

*Therefore, the sample of Row No. 4 in Table 9 was selected as the optimal sample.*

Page 22

In section 3.2 authors state that "As mentioned in the third chapter, the sample is closed by 6 screws at a distance of one centimeter from the beginning and the end of the sample." But there is no other place in the document where screws are mentioned. Moreover, can the authors clarify the meaning of "Therefore, this part of the sample is considered for closing fixed screws."

Answer: The respected reviewer is generally correct. In order to address this comment, the text was changed to the following sentences,

*The sample was closed by 6 screws at a distance of 1 cm from the beginning and the end part of the sample.* *Therefore, this part of the specimen was freeze. In fact, the geometric constraint of freezing was considered for this part of the sample.*

Page 26

Can the authors clarify what is the meaning of a “0.7 lifetime” in the sentence “According to the results, in general, it can be said that the normal lifetime of TO and SC samples is the same on average and is around 0.7.”

Answer: With respect, in this sentence, the normalized lifetime of the samples is discussed according to Figure 19. Therefore, this figure and sentence was changed to the following parts,

*According to the results in Figure 14, in general, it can be said that the normalized lifetime of TO and CS samples is the same on average and is around 0.7.*

The mentioned value is also highlighted in Figure 14, as follows,

Figure 14: The Pareto chart for the specimens under fatigue testing

Images of Table 21 are not with a proper resolution.

Answer: Table 21 was changed to the following one.

Table 21: Different conditions on the compressive test

| Sample Name | Test conditions | Num. |
| --- | --- | --- |
| Solid1, Solid2, Solid3 |  | 1 |
| Meta1, Meta2, Meta3 |  | 2 |
| Meta4, DLP |  | 3 |

The sentence “These samples are designed for use in car tires. Manufacturers are always looking for lower weight in the car. Since the lighter car weight brings benefits, such as reducing fuel consumption and emissions. Therefore, if the designer is looking for lightweight design of NPTs, he can use this type of cell.”

This is a clear example of a paragraph that need to be English revised. Also, this sentence should be relocated, potentially to the final conclusions or introduction, since similar content in these sections of the document already exist.

Answer: Thanks for your nice comment. The mentioned sentence was changed to the following one and it is relocated in the conclusion part, as follows,

- *Cellular structures (CS) with 15% of lower weight than the optimized samples had the same fatigue lifetime (0.7 of the normal lifetime). These samples were designed for the application in car tires. Manufacturers are always looking for lower weight in the car. Since the lighter car brings benefits, such as reducing the fuel consumption and emissions. Therefore, if the designers are seeking for the lightweight design of NPTs, they could use this cell type.*

Page 31

Figure 23 could be improved. “Defect caused by manufacturing method” is not a conclusive cause of defects. It is not clear what was the printing orientation of the specimens. Please add a comment, and characterize the different defects.

Answer: With respect to the opinion of the reviewer, this sentence was from the revised manuscript.

In addition, the print orientation and other defects were showed in Figure 17.

Figure 17: FE-SEM images for the fractured surfaces of PLA sample under tensile loading

Page 33

Could the authors verify the first bullet according with a previous comment? Also, the same bullet says 4.2 mm, is this the same conclusion of figure 1?

Answer: Yes, that is correct and the first bullet in the conclusions is related to Figure 1 (the 3D printing capability). It is not related to the defects of 3D printing. In order to address this comment, the first bullet is changed as follows,

- *According to the capability of FFF 3D printing, the smallest HC unit cell was 4.2 mm for the length.*
